# Molecular basis of sugar recognition by collectin-K1 and the effects of mutations associated with 3MC syndrome

**DOI:** 10.1186/s12915-015-0136-2

**Published:** 2015-04-17

**Authors:** Umakhanth Venkatraman Girija, Christopher M Furze, Alexandre R Gingras, Takayuki Yoshizaki, Katsuki Ohtani, Jamie E Marshall, A Katrine Wallis, Wilhelm J Schwaeble, Mohammed El-Mezgueldi, Daniel A Mitchell, Peter CE Moody, Nobutaka Wakamiya, Russell Wallis

**Affiliations:** Department of Infection, Immunity and Inflammation, University of Leicester, Leicester, LE1 9HN UK; Department of Biochemistry, University of Leicester, Leicester, LE1 9HN UK; Department of Medicine, University of California San Diego, La Jolla, CA 92093-0726 USA; Department of Microbiology and Immunochemistry, Asahikawa Medical University, 2-1-1-1 Midorigaoka-Higashi, Asahikawa, 078-8510 Japan; Department of Applied Science and Health, Coventry University, Coventry, CV1 5FB UK; Clinical Sciences Research Laboratories, Warwick Medical School, University Hospital Coventry & Warwickshire Coventry, Coventry, CV2 2DX UK

**Keywords:** Complement activation, structural biology, C-type lectin, 3MC syndrome

## Abstract

**Background:**

Collectin-K1 (CL-K1, or CL-11) is a multifunctional Ca^2+^-dependent lectin with roles in innate immunity, apoptosis and embryogenesis. It binds to carbohydrates on pathogens to activate the lectin pathway of complement and together with its associated serine protease MASP-3 serves as a guidance cue for neural crest development. High serum levels are associated with disseminated intravascular coagulation, where spontaneous clotting can lead to multiple organ failure. Autosomal mutations in the CL-K1 or MASP-3 genes cause a developmental disorder called 3MC (Carnevale, Mingarelli, Malpuech and Michels) syndrome, characterised by facial, genital, renal and limb abnormalities. One of these mutations (Gly^204^Ser in the CL-K1 gene) is associated with undetectable levels of protein in the serum of affected individuals.

**Results:**

In this study, we show that CL-K1 primarily targets a subset of high-mannose oligosaccharides present on both self- and non-self structures, and provide the structural basis for its ligand specificity. We also demonstrate that three disease-associated mutations prevent secretion of CL-K1 from mammalian cells, accounting for the protein deficiency observed in patients. Interestingly, none of the mutations prevent folding or oligomerization of recombinant fragments containing the mutations *in vitro*. Instead, they prevent Ca^2+^ binding by the carbohydrate-recognition domains of CL-K1. We propose that failure to bind Ca^2+^ during biosynthesis leads to structural defects that prevent secretion of CL-K1, thus providing a molecular explanation of the genetic disorder.

**Conclusions:**

We have established the sugar specificity of CL-K1 and demonstrated that it targets high-mannose oligosaccharides on self- and non-self structures via an extended binding site which recognises the terminal two mannose residues of the carbohydrate ligand. We have also shown that mutations associated with a rare developmental disorder called 3MC syndrome prevent the secretion of CL-K1, probably as a result of structural defects caused by disruption of Ca^2+^ binding during biosynthesis.

**Electronic supplementary material:**

The online version of this article (doi:10.1186/s12915-015-0136-2) contains supplementary material, which is available to authorized users.

## Background

Collectin-K1 (CL-K1) is a multifunctional secreted pattern-recognition lectin with key roles in host defence, tissue homeostasis and embryogenesis [1,2]. It binds to pathogen-associated molecular patterns and activates mannan-binding lectin (MBL)-associated serine proteases (MASPs-1, −2 and −3) to initiate the lectin pathway of complement activation, and stimulate immune and inflammatory processes [3,4]. It also binds to apoptotic cells and may function as a scavenger receptor to facilitate their clearance as has been shown for other collectins. Elevated serum levels are associated with a severe clotting disorder called disseminated intravascular coagulation, characterised by systemic coagulation and microvascular thrombi, that often leads to multiple organ failure [5]. During embryogenesis, CL-K1 is highly expressed in craniofacial cartilage, heart, bronchi, kidney, and vertebral bodies and together with MASP-3 probably serves as a guidance cue for neural crest cell migration by recognising specific endogenous carbohydrate epitopes [6]. Separate autosomal mutations in the genes encoding CL-K1 or MASP-3 lead to a rare autosomal recessive disorder called Carnevale, Mingarelli, Malpuech and Michels (3MC) syndrome, associated with craniofacial dysmorphism, mental retardation and genital, renal and limb abnormalities, highlighting the importance of these complexes during development.

CL-K1 is a member of the collectin family of animal lectins with an N-terminal collagen-like domain linked to C-terminal carbohydrate-recognition domains (CRDs) via a helical neck [7]. The collagenous stems are tethered together at the N-terminus to form characteristic bouquet- or fan-shaped structures. CL-K1 can form homooligomers as well as heterooligomers with the related molecule collectin-L1 (CL-L1) [8]. Clusters of CRDs recognise their carbohydrate targets through multiple weak interactions [9]. Ca^2+^ plays an essential role in this process by forming a ternary complex with the protein and the glycan ligand and by stabilising the CRDs [10-12]. CL-K1 binds to a variety of intact bacteria, fungi, and viruses including *Escherichia coli*, *Candida albicans* and Influenza A virus [3]. It also binds to DNA, explaining at least in part how it targets apoptotic cells [13]. Although selective for mannose and fucose, CL-K1 binds only weakly to monosaccharides (IC_50_ approximately 20 mM) compared to MBL (*K*_*D*_ approximately 1 mM) [14] and other collectins, and little is known about its specificity towards oligosaccharides on self- or non-self structures [3].

Three separate disease-associated mutations have been identified in the coding region of the CL-K1 gene. All lead to changes in the primary structure of the CRD: two result in single amino acid substitutions: Ser^169^Pro and Gly^204^Ser and the third leads to the deletion of Ser^217^. CL-K1 is undetectable in the serum of individuals homozygous for the Gly^204^Ser mutation, implying that it is either not secreted or is degraded in serum. However, the other disease-associated mutations have not been characterised [6].

Here we show that CL-K1 primarily recognises a subset of high-mannose oligosaccharides containing the disaccharide motif: Man(α1-2)Man found on both self and non-self structures. It binds to both sugar moieties, in contrast to other collectins that only recognise the terminal sugar. We also show that all three mutations associated with 3MC syndrome prevent normal secretion from mammalian cells probably as a result of structural changes caused by the failure to bind Ca^2+^ during biosynthesis. The protein deficiency would prevent the normal recognition processes of CL-K1/MASP-3 complexes during development, leading to the 3MC phenotype.

## Results

### Sugar specificity of CL-K1 towards self and non-self ligands

**Figure 1:**
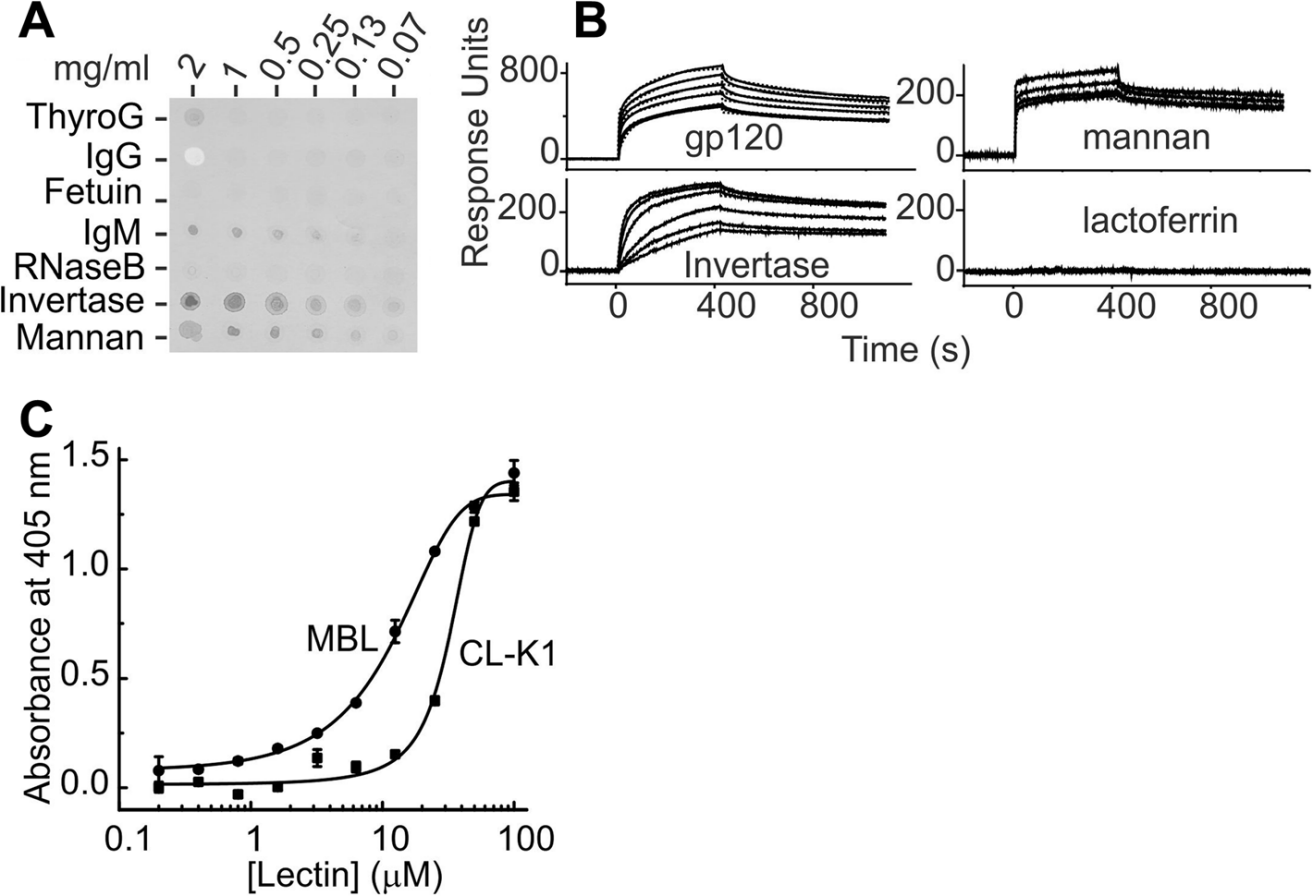
Glycoprotein binding and complement activation by CL-K1. **A)** Immunoblot of CL-K1 binding to immobilised mammalian and yeast glycoproteins. **B)** Surface plasmon resonance of CL-K1 binding to glycoproteins. CL-K1 was immobilised on the chip surface (6,000 response units) and six two-fold dilutions of each glycoprotein were injected at a starting concentration of 200 nM. **C)** Complement component C3b was measured on mannan following incubation with human serum, depleted of endogenous lectins, and supplemented with either CL-K1 or human recombinant MBL. C3b and its breakdown products were detected by absorbance using an anti-human C3c antibody with alkaline phosphatase-conjugated goat anti-rabbit secondary antibody and p-nitrophenyl phosphate as the substrate. Protein concentrations were calculated based on the molecular masses of CL-K1 and MBL trimers (78 kDa in each case). CL-K1, collectin-K1; MBL, mannan-binding lectin.

To characterise the sugar specificity of CL-K1, we initially screened a selection of glycoproteins with well-characterised glycans (Figure 1A). Blot analysis revealed binding to immunoglobulin M (IgM), thyroglobulin and yeast invertase and mannan, but not to RNAse B, fetuin or IgG. The most discernible difference in the pattern of recognition was the presence of high-mannose oligosaccharides on the glycoproteins that were recognised, compared to predominantly complex sugars on those that were not. An exception was RNAse B, which possesses a single N-linked glycosylation site, mainly occupied by Man_5_ (approximately 60%) but with some Man_6_-Man_9_ [15]. To explore carbohydrate binding further, the binding kinetics was determined for yeast invertase, mannan and gp120 from HIV that all possess high-mannose oligosaccharides. CL-K1 bound tightly to all three glycoproteins, but not to milk lactoferrin that contains complex sugars (Figure 1B). In each case, the data fitted best to a two-complex model and the kinetic parameters are presented in Table 1. To confirm that carbohydrate binding leads to activation of complement, C3b deposition was measured on mannan in whole human serum depleted of endogenous lectins (Figure 1C). CL-K1 activated complement with only 2.5-fold lower activity than human MBL.

**Table 1:** **Kinetics of binding of CL-K1 to glycoproteins**

**Glycoprotein**	***k***_***on***_	***k***_***off***_	***K***_***D***_
	**(M** ^**−1**^ **s** ^**−1**^ **)**	**(s** ^**−1**^ **)**	**(nM)**
Mannan	1.6 ± 0.3 × 10^3^	<1.0 × 10^−6^	<0.8
	1.0 ± 0.2 × 10^6^	3.0 ± 0.2 × 10^−4^	0.31 ± 0.9
Invertase	6.8 ± 0.4 × 10^4^	1.2 ± 0.2 × 10^−3^	18 ± 4
	6.7 ± 0.5 × 10^5^	<1.0 × 10^−6^	<0.002
gp120	1.5 ± 0.3 × 10^5^	1.1 ± 0.2 × 10^−3^	8 ± 4
	>1 × 10^6^	<1.0 × 10^−6^	<0.001

All data were fitted to a two-complex binding model. Values of *k*_*on*_ above 1 × 10^6^ M^−1^ s^−1^ and *k*_*off*_ below 1.0 × 10^−6^ s^−1^ exceed the reliable detection limits of the instrument, so are given as lower and upper limits respectively. Data are mean ± error from two experiments.

**Figure 2:**
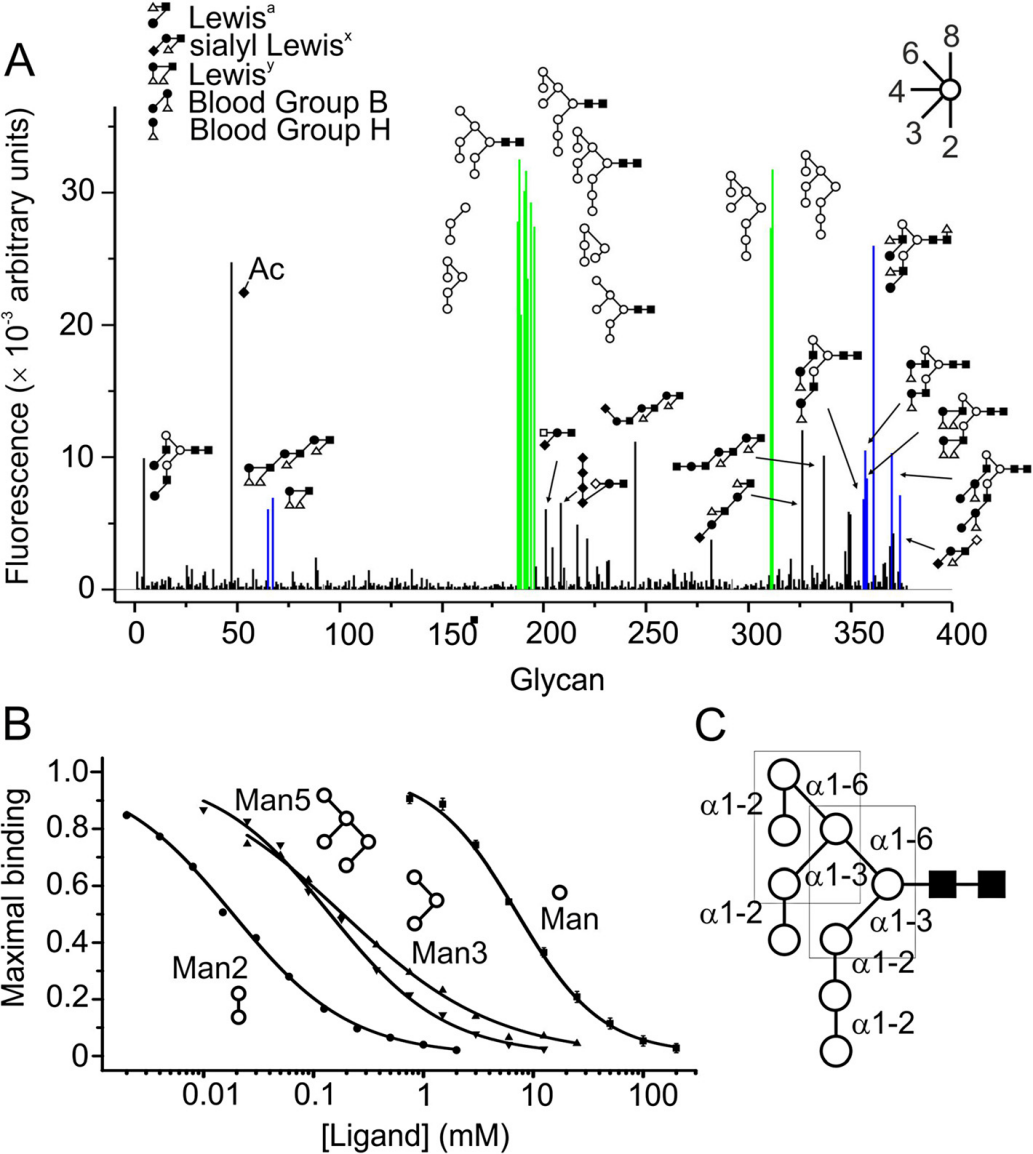
Carbohydrate binding by CL-K1. **A)** Glycan-array analysis of CL-K1. Key: open circle, mannose; closed circle, galactose, closed square, N-acetylglucosamine; closed diamond, N-acetylneuraminic acid; open square, N-acetylgalactosamine; open diamond, glucose; and triangle, fucose. High-mannose and blood group/Lewis antigen ligands are in green and blue. **B)** Inhibition of CL-K1 binding to immobilized yeast invertase (10 μg per well) by high-mannose epitopes (Man2: α1-2 mannobiose; Man3: α1-3, α1-6 mannotriose; Man5: α1-3, α1-3, α1-6 Mannopentaose). Values are the mean ± error from duplicate measurements. **C)** Schematic representation of the mammalian high-mannose glycan (Man_9_GlcNAc_2_). Boxes show the two Man_3_ core structures that together form the Man_5_ core. CL-K1, collectin-K1.

To examine binding to individual glycans, fluorophore-labelled CL-K1 was used to probe a broad screen of mammalian carbohydrates using the Core H Glycan array technology facility at The Consortium for Functional Glycomics [16]. The array consisted of 377 natural and synthetic mammalian glycans attached to the surface of a glass microscope slide via covalent amide linkages. The relative fluorescence signal provides an accurate indication of the order of affinities [17]. Consistent with the glycoprotein data, the best ligands were all high-mannose oligosaccharides (Figure 2A and Additional file 1), but not all of these were recognised. Mammalian high-mannose structures comprise a Man_5_ core attached to the protein via two GlcNAc residues, with α1-2 linked sugars at the non-reducing termini (Figure 2C). CL-K1 only recognised those structures containing at least one terminal Man(α1-2)Man epitope. For example, it bound tightly to Man_6,_ Man_7_, Man_8_ and Man_9_ but not to the Man_5_ core (ligand 310) that comprises only α1-3 and α1-6 linkages. It also bound to smaller glycans containing the essential disaccharide epitope, for example, the trisaccharide, Man(α1-2)Man(α1-3)Man (ligand 189). The GlcNAc residues were neither necessary nor enhanced binding (for example, compare Man_9_GlcNac_2_, ligand 192, and Man_9_, ligand 312). This pattern of recognition is different from other collectins, such as MBL and pulmonary-surfactant protein D that typically recognise the terminal sugar alone (mannose, fucose or N-acetyglucosamine) [18,19]. It is also different from other C-type lectins, such as DC-SIGN or DC-SIGNR that target larger high-mannose oligosaccharides preferentially (Man_5_ to Man_9_) [17]. To quantify the differences in affinities, we compared binding to three different epitopes that all form part of a high-mannose oligosaccharide. Inhibition by Man(α1-2)Man (IC_50_ of 0.02 mM) was 10- and 8-fold greater than for the Man_3_ and Man_5_ core structures, and >300-fold greater than for mannose alone (Figure 2C). Thus, CL-K1 primarily recognises the terminal disaccharide of the glycan, rather than the terminal sugar alone, explaining its unusual binding properties.

CL-K1 also bound to subsets of fucosylated glycans, including those containing Lewis^a^, Lewis^y^ and blood group B antigens as well as to a selection of structures containing blood group H antigens (Figure 2A). Most fucosylated sugars were not recognised, however, suggesting that CL-K1 contacts multiple monosaccharide moieties in each glycan and that the chemical environment of the binding epitope is important. Binding to almost all ligands was Ca^2+^-dependent (see Additional file 1), compatible with the pivotal role of Ca^2+^ in carbohydrate recognition by C-type lectins. An exception was 9-O-acetylneuraminic acid, which gave a similar binding signal in the presence and absence of Ca^2+^, implying that it interacts with a distinct and independent binding site on CL-K1.

### Structure of CL-K1 in complex with a disaccharide ligand

**Figure 3:**
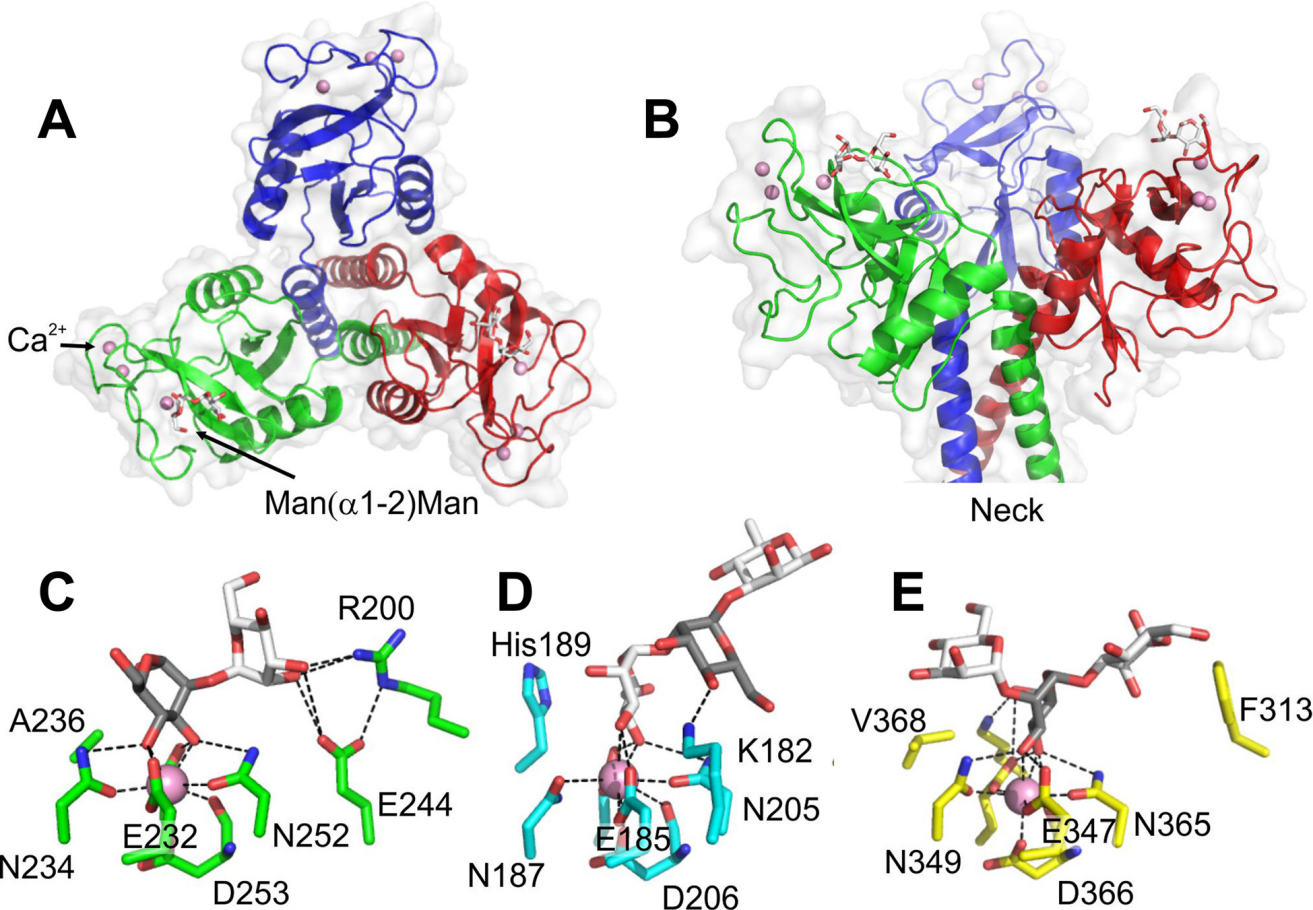
Structure of a trimeric fragment of CL-K1 bound to a mannose disaccharide. **A** and **B)** top and side views. The carbohydrate is in white and Ca^2+^ in pink*.* Comparison of carbohydrate binding by **C)** CL-K1; **D)** mannan-binding lectin (PDB: 1KWY) and **E)** DC-SIGN (PDB: 2IT5). Polar interactions are shown by dotted lines. The penultimate sugar moiety (internal in a high-mannose oligosaccharide) is in grey. CL-K1, collectin-K1.

In order to determine the precise specificity and mechanism of carbohydrate recognition by CL-K1, a recombinant trimeric fragment comprising the neck and CRDs was produced in *E. coli*, refolded from inclusion bodies and was crystallized in the presence of Ca^2+^, and structures were determined both with and without its primary ligand, Man(α1-2)Man (PDB: 4YLI and 4YMD for the CL-K1 trimer and the trimer bound to Man(α1-2)Man; Table 2). The structures confirm that the CL-K1 fragment is a homotrimer with the three CRDs linked via an α-helical coiled coil (Figure 3). The asymmetric unit of each crystal contained two trimers with each CRD bound to three Ca^2+^. Density for the sugar was present for four of the six polypeptides (Figure 3A and B). In the ligand complex, the reducing mannose of the disaccharide binds to the Ca^2+^ common to all C-type lectins, with the equatorial 3- and 4-OHs of the sugar forming coordination bonds with the Ca^2+^ and hydrogen bonds to surrounding Asn, Asp and Glu residues that also serve as Ca^2+^ ligands. The interaction is unusual, however, because it involves the penultimate sugar (internal in a high-mannose oligosaccharide) rather than the terminal sugar (non-reducing) that is recognised by other collectins (Figure 3C and D) [10,19]. In addition, the terminal mannose forms hydrogen bonds to the carboxylate group of Glu^244^ and the guanidinium group of Arg^200^ to form a small hydrogen-bonding network that stabilises the interaction compared to mannose alone, providing an explanation for its binding specificity. Comparison of the bound and unbound structures reveals no major conformational changes upon carbohydrate recognition. The interaction is somewhat reminiscent of glycan binding by DC-SIGN and DC-SIGNR, which also target high-mannose oligosaccharides via internal sugar moieties (Figure 3E). The ligand specificities of CL-K1 and DC-SIGN/DC-SIGNR are significantly different, however, as noted above.

**Table 2:** **Data collection and refinement statistics**

**Structural data**	**CL-K1 trimer**	**CL-K1 + Man**_**2**_
**PDB code**	4YLI	4YMD
**Data collection**		
**Beamline**	Diamond I04	Diamond I04-1
**Space group**	P 1 2_1_ 1	P 1 2_1_ 1
**a, b, c, Å**	73.0 107.6 77.8	71.9 106.7 78.4
**α, β, γ, °**	90, 91.5, 90	90, 92.2, 90
**Resolution, Å**	48.21-2.45 (2.538-2.45)	44.08-2.87 (2.973-2.87)
**R**_**sym**_	0.054 (0.48)	0.052 (0.54)
**I/σI**	10.1 (2.0)	11.2 (1.8)
**Completeness, %**	95.5 (87.2)	91.0 (93.6)
**Redundancy**	3.3 (3.1)	2.9 (2.9)
**Refinement**		
**Resolution, Å**	48.21-2.45 (2.538-2.45)	44.08-2.87 (2.973-2.87)
**Unique reflections**	42224 (3791)	24670 (2518)
**R**_**work**_ **/R**_**free**_	0.1869/0.2212	0.2129/0.2607
**No of atoms**	7332	7220
**Protein**	7162	7062
**Ligands/ion**	121	152
**water**	49	6
**B-factors, Å** ^**2**^	74.5	93.2
**macromolecules**	74.5	93.0
**ligands**	76.4	105.6
**water**	60.7	62.6
**rms deviations**		
**Bond lengths, Å**	0.003	0.003
**Bond angles, °**	0.62	0.77

The highest-resolution shell is shown in parentheses.

### Ca^2+^ stabilises CL-K1 by organising the binding loops of the CRDs

**Figure 4:**
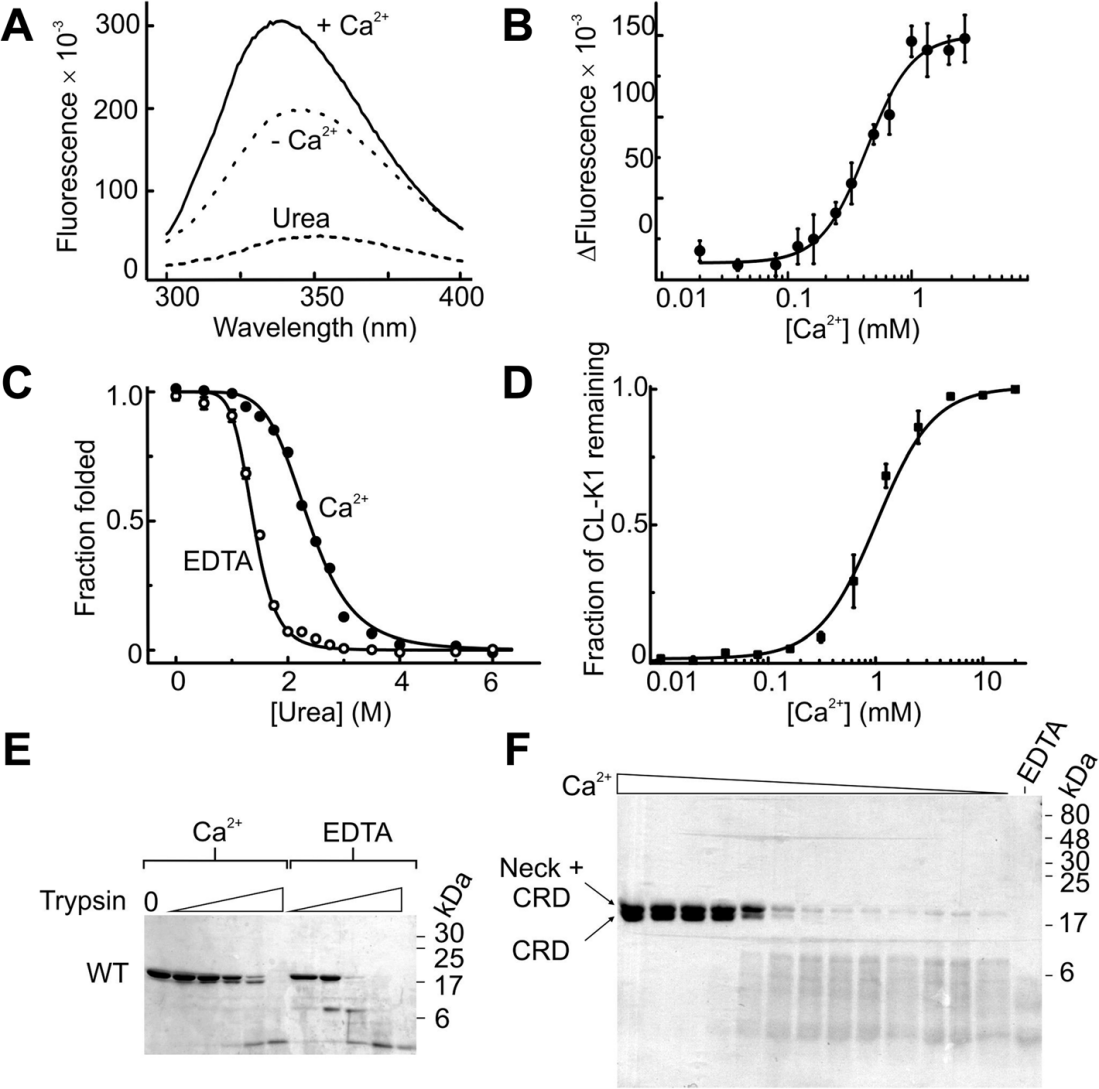
Ca^2+^ stabilises the CRDs of CL-K1. **A)** Fluorescence spectra of the CL-K1 fragment in different buffers. The λ_ex_ was 280 nm. **B)** Ca^2+^ binding to the CL-K1 fragment measured by fluorescence. Measurements were recorded at an λ_em_ of 345 nm. **C)** Urea denaturation in the presence and absence of Ca^2+^ (2 mM). **D)** Proteolysis of CL-K1 in the presence of increasing concentrations of Ca^2+^ (two fold dilutions starting at 5 mM). Samples were separated by SDS-PAGE and gels were scanned to determine the amount of CL-K1 remaining. The amount of trypsin was 0.5% (w/w). The intensities of bands corresponding to the neck and CRD and the CRD alone were combined. A sample gel is shown in **F)**. **E)** Proteolysis of CL-K1 in the presence and absence of Ca^2+^. Two fold serial dilutions of trypsin were used at a starting concentration of 2% (w/w). In all experiments data are mean ± error from two separate experiments. CL-K1, collectin-K1; CRD, carbohydrate-recognition domain.

Ca^2+^ is integral to the structure and function of CL-K1 (Figure 3). It not only interacts directly with the carbohydrate ligand but also binds to loops at the top of the CRD. We used the intrinsic fluorescence of the trimeric fragment as a probe to measure Ca^2+^ binding. Each CRD contains two tryptophan residues, which together form part of the hydrophobic core and are sensitive to changes in the conformation of the adjacent Ca^2+^-binding loops. Addition of Ca^2+^ caused a large enhancement of the native fluorescence (approximately 50%) combined with a shift in λ_em, max_ to a shorter wavelength (from 346 nm to 339 nm), as the tryptophans move to a more hydrophobic environment (Figure 4A). The *K*_*D*_ was measured as 0.43 mM by titration with Ca^2+^, with a Hill coefficient of 2.4 (Figure 4B). Thus, although only one Ca^2+^ interacts with the ligand directly, binding itself is highly cooperative, with all three Ca^2+^ being important.

We also investigated the role of Ca^2+^ towards the stability of the CRDs of CL-K1 using urea denaturation. Unfolding of the CRD/neck fragment was accompanied by a dramatic quenching of the fluorescence and a shift in λ_em, max_ to approximately 354 nm as the tryptophans become exposed to the polar solvent (Figure 4A). Unfolding could be explained by a simple two-state transition, both in the presence and absence of Ca^2+^. However, the midpoint (denaturation concentration) was 2.4 M and 1.4 M urea for the Ca^2+^-bound and -free forms (Figure 4C), indicating that Ca^2+^ stabilises the CRDs.

**Figure 5:**
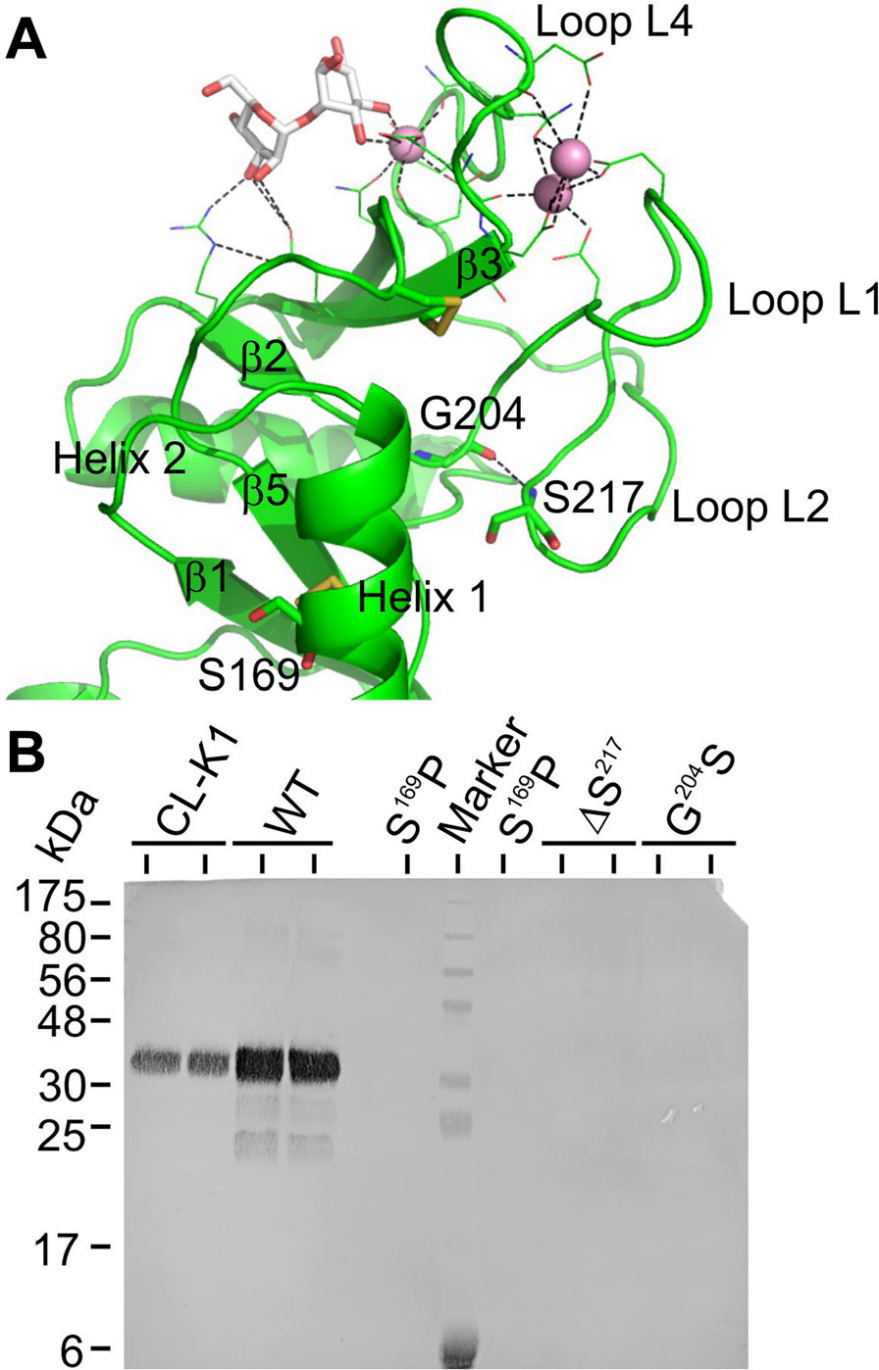
No secretion of variant CL-K1s containing mutations associated with 3MC syndrome. **A)** Structure of the CRD of CL-K1 showing the position of mutations associated with 3MC syndrome. Secondary structure features are labelled based on the structure of MBL [38]. Polar interactions are indicated by dotted lines. **B)** Secretion of wild type and variant CL-K1s from mammalian cells. Serum-free medium from Chinese hamster ovary cells (20 μL) transfected with wild-type (WT) or variant cDNAs was separated on a 15% SDS-PAGE gel under non-reducing conditions and blotted onto a nitrocellulose membrane. Secreted CL-K1 was detected using rabbit polyclonal anti-CL-K1 antibodies. Purified wild-type CL-K1 (approximately 20 ng) was used as a positive control. CL-K1, collectin-K1; CRD, carbohydrate-recognition domain; MBL, mannan-bindng lectin; 3MC, Carnevale, Mingarelli, Malpuech and Michels.

The three Ca^2+^ interact with residues primarily from loops towards the top of the CRD (Figure 5A). In the absence of Ca^2+^, these regions are likely to be more flexible. To investigate this possibility, we compared the susceptibility of the trimeric CL-K1 fragment towards trypsin, which preferentially cleaves disordered regions of proteins. The Ca^2+^-free form was highly sensitive to trypsin (Figure 4E). By contrast, an approximately 15 kDa fragment was generated in the presence of Ca^2+^ that was relatively resistant to further proteolysis (Figure 4E and F). This fragment was identified as the CRD by Edman degradation, beginning at residue Glu^148^. By measuring proteolysis in the presence of increasing concentrations of Ca^2+^, the apparent *K*_*D*_ for calcium was determined as approximately 1 mM, in good agreement with fluorescence data (Figure 4D and F). Thus, Ca^2+^ stabilises the CRD by tethering the binding loops together. In the absence of Ca^2+^, these loops become disordered and more exposed.

### Mutations associated with 3MC syndrome prevent secretion of CL-K1

As well as explaining the mode of carbohydrate recognition, the structures of CL-K1 also reveal the location of residues that are altered in the 3MC syndrome (Ser^169^, Gly^204^ and Ser^217^). Gly^204^ and Ser^217^ are located at the base of loop L1 (Figure 5A) that forms part of the binding site for two of the three Ca^2+^. The carbonyl group of Gly^204^ forms a hydrogen bond with the amide of Ser^217^, helping to anchor the loop to the side of the domain. Ser^169^ forms part of one of two helices at the base of the CRD, close to the helical neck of the trimer.

To characterise the disease-associated mutations, corresponding changes were introduced into the cDNA of CL-K1, and proteins were expressed in Chinese hamster ovary cells. Immunoblotting of media from transfected cells revealed that although wild-type CL-K1 was secreted as expected, no protein was detected in the media of cells transfected with any of the mutant cDNAs (Figure 5B), implying that the encoded proteins are not secreted. This conclusion would explain the protein deficiency in individuals with the Gly^204^Ser mutation and suggests that those individuals with Ser^169^Pro or ΔSer^217^ mutations are also likely to be deficient in CL-K1.

### Defective Ca^2+^ binding by CL-K1 containing disease-associated mutations probably leads to elimination during biosynthesis

Surprisingly, when recombinant fragments of CL-K1 (neck and CRDs) containing the mutations (subsequently referred to as variant fragments) were produced in *E. coli*, they refolded like the wild-type fragment, and good yields of purified protein were isolated (1 to 2 mg/L of culture). This finding was unexpected because we anticipated that the mutations would severely destabilise the CRDs, thus preventing folding. It was therefore of interest to characterise these fragments in more detail to attempt to understand why the mutations are deleterious and prevent secretion *in vivo*.

**Figure 6:**
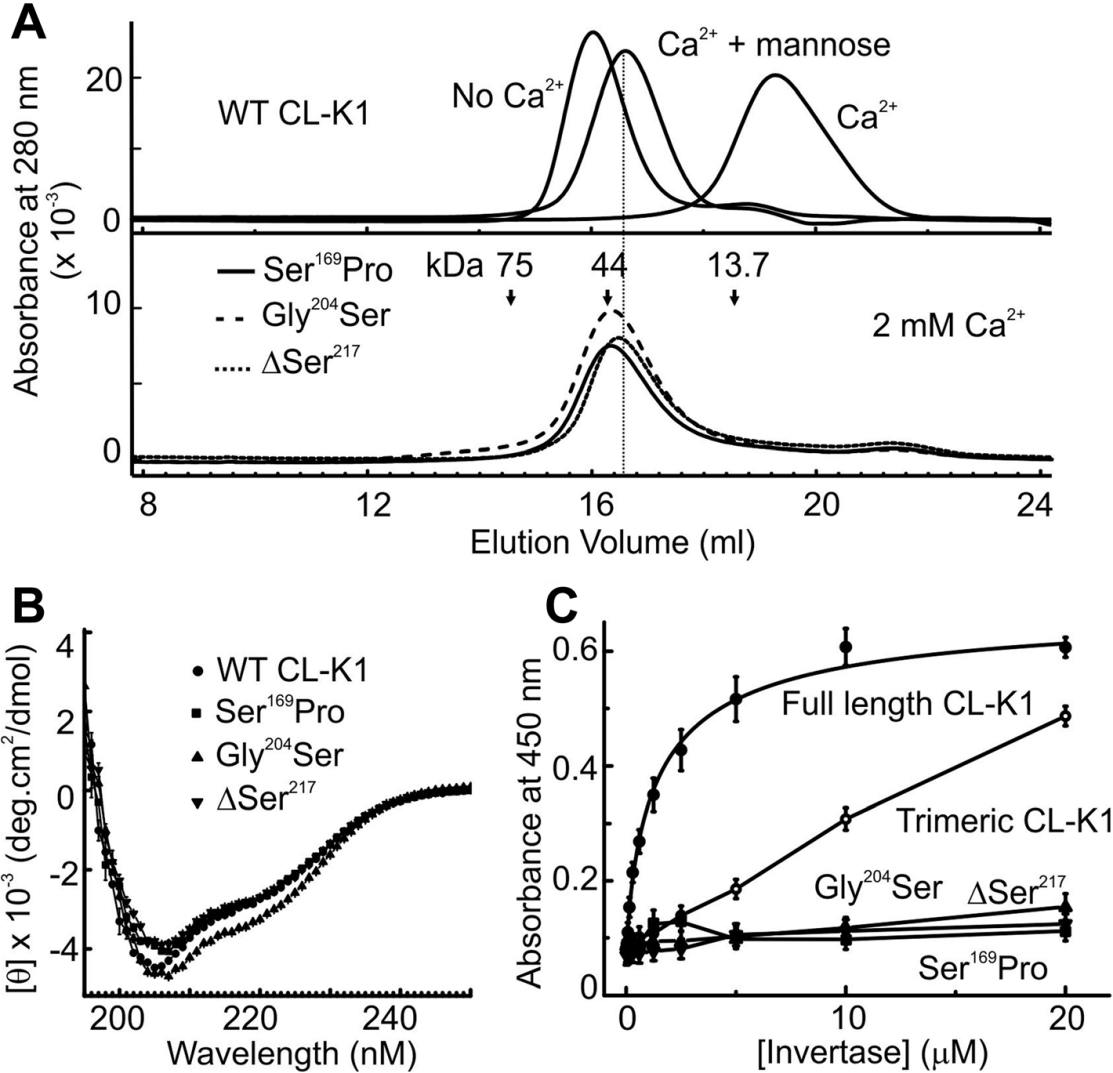
Characterisation of CL-K1 fragments containing disease-associated mutations. **A)** Gel filtration on a Superdex 200 (10/30 column) in 50 mM Tris–HCl at pH 7.4, containing 150 mM NaCl. The elution positions of conalbumin (75 kDa), ovalbumin (44 kDa) and ribonuclease A (13.7 kDa) are indicated. Top, The wild-type CL-K1 fragment interacts with the column matrix in buffer containing Ca^2+^ (2 mM) but not in the absence of Ca^2+^ (2 mM EDTA) or in the presence of inhibitory concentrations of mannose (200 mM). Bottom, None of the three CL-K1 variants interacts with the column, even in the presence of Ca^2+^. **B)** CD spectra of the wild-type CL-K1 fragment and the disease-associated CL-K1 variants. **C)** Binding of biotinylated invertase to full length CL-K1 and trimeric fragments. Values are the mean ± error from duplicate measurements. Tighter binding of the full-length protein is due to its increased avidity. CL-K1, collectin-K1; EDTA, ethylenediaminetetraacetic acid.

**Figure 7:**
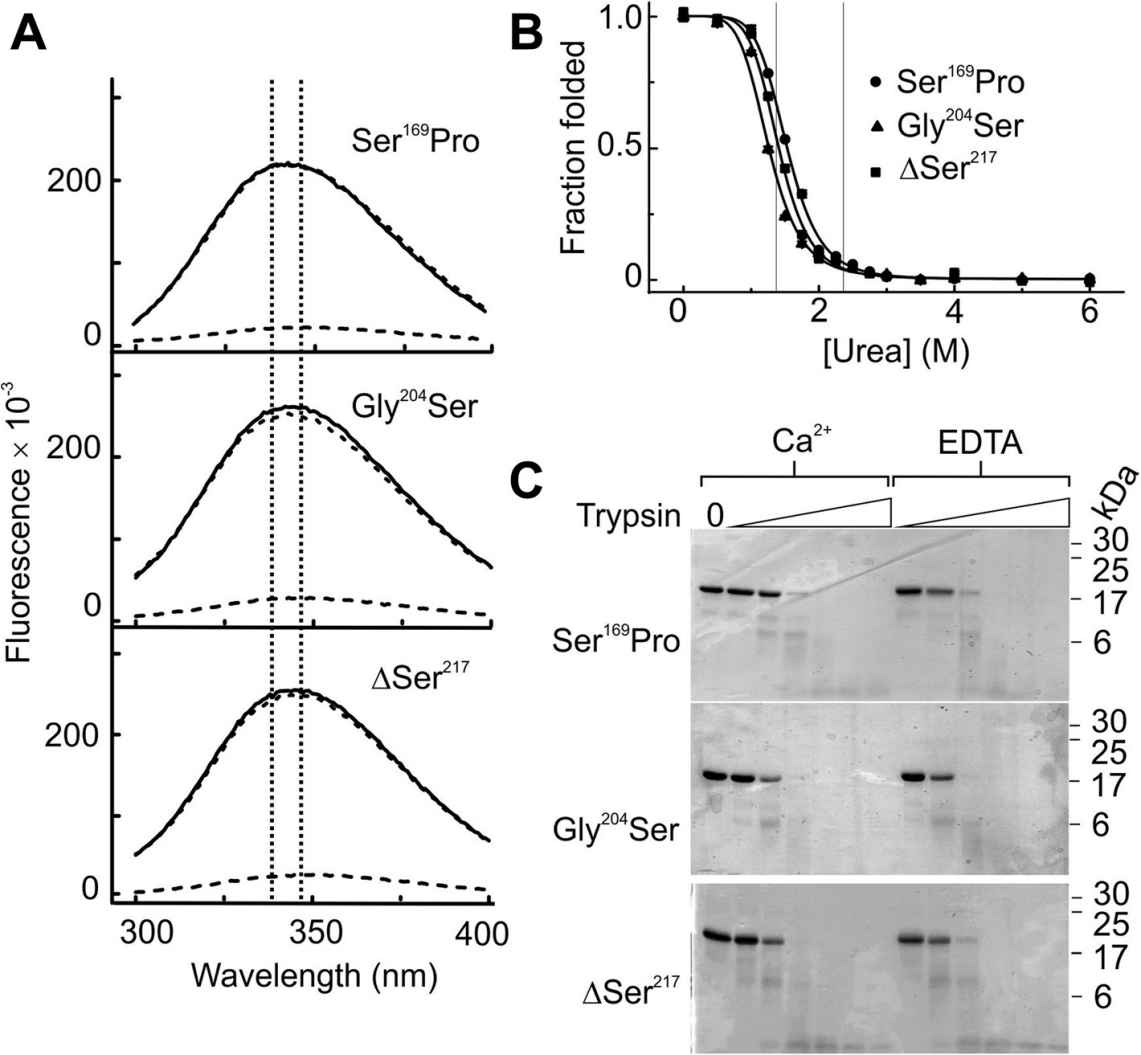
Destabilisation of variant CL-K1s caused by the failure to bind to Ca^2+^. **A)** Fluorescence spectra of variant CL-K1 trimers in the presence (solid line, 2 mM Ca^2+^) and absence of Ca^2+^ (dotted line, 2 mM EDTA) and in 6 M urea (dashed line). The λ_em, max_ of Ca^2+^ bound and Ca^2+^ free forms of the wild-type fragment are indicated by vertical dotted lines. **B)** Unfolding of the variant CL-K1 (lower) fragments in urea. All experiments were performed in the presence of 2 mM Ca^2+^ and data were recorded at 345 nM. Values are mean ± error from duplicate experiments. The denaturation concentrations of Ca^2+^-bound and Ca^2+^-free forms of the wild-type fragment are indicated by vertical dotted lines. **C)** SDS-PAGE gels (17.5%) showing proteolysis of variant CL-K1 fragments with two-fold serial dilutions of trypsin (highest concentration is 4% w/w). CL-K1, collectin-K1; EDTA, ethylenediaminetetraacetic acid.

Collectins normally fold in the endoplasmic reticulum (ER), in the presence of Ca^2+^ where resident chaperones monitor the folding status of proteins [20]. Misfolded or partially folded proteins with exposed hydrophobic regions are recognised by these chaperones, and are targeted for elimination via the ER-associated protein degradation pathway [21]. Consequently, any structural defects caused by the mutations are likely to prevent secretion. Circular dichroism indicated that all three variants were folded, with spectra characteristic of the α/β structure of the neck + CRD regions (Figure 6B). This finding was confirmed by fluorescence spectroscopy and urea denaturation experiments (Figure 7A and B; described in more detail below). The first clue that they were defective was from gel filtration analysis. Like many lectins, CL-K1 binds to dextran, so is retarded on gel filtration columns such as Superdex. This binding could be prevented by excess mannose as a competitive inhibitor, or by ethylenediaminetetraacetic acid (EDTA) that chelates the Ca^2+^ (Figure 6A), whereupon the wild-type fragment eluted at the position of a homotrimer (47 kDa). The variant fragments were also trimers, but did not bind to the dextran matrix even in the presence of Ca^2+^, implying loss of carbohydrate binding. To confirm this possibility, we measured binding to yeast invertase. Both full-length CL-K1 and the wild-type fragment bound to the invertase, but the three variant fragments did not bind (Figure 6C). Thus, although folded and assembled into trimers, the mutations disrupt the CRDs preventing normal interactions with carbohydrate ligands.

The most likely reason for the failure to bind carbohydrate is loss of Ca^2+^ binding. As described above, failure to bind Ca^2+^ destabilises the CRDs and exposes flexible loop regions. The presence of such defects during biosynthesis would likely lead to elimination of CL-K1 polypeptides via ER resident chaperones. We therefore tested Ca^2+^ binding using fluorescence assays described above. Addition of Ca^2+^ to the variant CL-K1s did not cause the changes in quantum yield or λ_em, max_ that were observed upon Ca^2+^ binding by the wild-type fragment (Figure 7A). Furthermore, urea denaturation curves resembled the Ca^2+^-free form, even in the presence of Ca^2+^, with denaturation concentrations of 1.5, 1.3 and 1.4 M for Ser^169^Pro, Gly^204^Ser, ΔSer^217^ proteins respectively (Figure 7B). Likewise, each of the variants was equally sensitive to trypsin in the presence or absence of Ca^2+^ (Figure 7B). Thus, all three mutations induce a common structural defect that arises due to the failure to bind to Ca^2+^. *In vivo*, such defects are likely to be detected by chaperones in the ER during biosynthesis leading to elimination. In this way, the mutations would prevent normal secretion of CL-K1, accounting for the protein deficiency in patients with 3MC syndrome.

## Discussion

The data described here explain for the first time how CL-K1 is able to target self- and non-self carbohydrate ligands to fulfil its diverse biological functions. Man(α1-2)Man epitopes are found at the non-reducing termini of many, but not all, mammalian high-mannose sugars. They are also found on fungal, viral and bacterial structures. For example, mannan from cell walls of *C. albicans*, a known target of CL-K1 [3], is rich in α1-2 linked mannose oligosaccharides [22]. Likewise, high-mannose oligosaccharides on viral haemagglutinin and gp120 enable CL-K1 to bind to Influenza A virus and HIV [3]. High-mannose oligosaccharides themselves represent only a small subset of mammalian glycans. Most glycoproteins and glycolipids display complex oligosaccharides that are based on a Man_3_GlcNac_2_ core not targeted by CL-K1, in which the two antennae are decorated with other sugars. The difference in specificity of CL-K1 compared to other collectins is due to the presence of an extended binding site on the CRD (Figure 3C). Whilst unusual for a collectin, additional interactions have been described for other C-type lectins including DC-SIGN, selectins and the macrophage receptor Mincle, each with different ligand preferences [23].

Heterocomplexes of CL-K1 and CL-L1 polypeptides may recognise additional ligands that are not targeted by CL-K1 homooligomers as a result of contacts mediated through the CRD of CL-L1. Like CL-K1, CL-L1 has a preference for ligands possessing mannose and/or fucose epitopes, although little is known about its specificity towards oligosaccharides on self or non-self structures. The presence of CL-K1/CL-L1 complexes as well as CL-K1 homooligomers in serum and in other tissue fluids may serve to increase the range of pathogens targeted by the lectin pathway. It is currently unclear, however, if CL-L1 participates in developmental processes or is involved in the aetiology of 3MC syndrome.

Ca^2+^ is essential for the structure and function of C-type lectins, because it stabilises the CRDs and helps position the loops in the correct arrangement for ligand binding. Although we were unable to crystallise CL-K1 in the absence of Ca^2+^, the structure of a Ca^2+^-free form of MBL reveals that the core of the CRD is largely unchanged but the loops are positioned differently with some regions being undetectable in the electron density, implying considerable flexibility [11]. A similar scenario for the CL-K1 variants would explain why the CD spectra are similar to wild type but the CRDs are much more sensitive to proteolysis.

Gly204 and Ser217 are located at the base of Ca^2+^-binding loop L1 (Figure 5A), so the disease-associated mutations probably interfere with the packing of this loop against the CRD, thereby preventing Ca^2+^ binding. The effects of the Ser^169^Pro mutation within Helix 1 are less clear. Nevertheless, the introduction of a proline residue in place of the serine is likely to destabilise the helix and preclude the normal interactions with the CRD. Notably, Tyr163 at the top of the helix forms part of the hydrophobic core and packs against residues in loop 1, forming a hydrogen bond with Asn206. Disruption of these contacts probably prevents Ca^2+^ binding, leading to the disease phenotype.

Folding of CL-K1, like other collectins, probably proceeds in a C- to N-terminal in which folding of the neck and CRDs nucleates assembly of the N-terminal collagen-like domains [20]. All three variant fragments folded and trimerised *in vitro*, so folding is still thermodynamically favourable, despite the mutations. It is thus likely that at least some polypeptides fold *in vivo*. Nevertheless, the CRDs will be defective with exposed, disordered regions, so variants are likely to be retained and subsequently eliminated by the ER-associated protein degradation pathway [21]. The possibility that the mutations prevent folding *in vivo* (but not *in vitro*) cannot be completely ruled out. However, this scenario would seem less likely, in particular because none of the mutations are greatly destabilising *per se* (Figure 7B). Instead, the decrease in stability in each case can be accounted for by the failure to bind to Ca^2+^.

The finding that CL-K1 binds to Lewis antigens and certain blood group antigens, including groups B and H, is both interesting and unexpected. Lewis y is expressed mainly during embryogenesis, and in adults expression is restricted to granulocytes and epithelial surfaces [24]. Elevated levels are also found in most epithelial-derived human carcinomas, including breast, ovary, prostate, colon cancers, and high levels are correlated with poor prognosis [24]. Platelets and coagulatory factors such as Von Willebrand Factor are all decorated with blood group antigens, and crosslinking of these components possibly leading to complement activation might account for the link between elevated CL-K1 serum levels and disseminated intravascular coagulation [25]. Notably, activation of the lectin pathway itself promotes activation of the clotting cascade potentially compounding the problem [26]. The larger binding pocket on CL-K1 and the resulting enhanced affinity for its glycan ligands compared to other collectins suggest that it may be possible to design inhibitors that selectively block ligand binding. These would have therapeutic potential for the treatment of disseminated intravascular coagulation and other disorders associated with profound activation of the innate immune and/or coagulation systems, such as in ischaemia-reperfusion injury.

## Conclusions

In conclusion, we have shown that CL-K1 binds to high-mannose oligosaccharides and fucosylated sugars including blood group antigens and Lewis antigens. It binds to high-mannose oligosaccharides via an extended binding site that recognises the terminal two mannose residues. In addition, we have shown that three naturally occurring mutations associated with a severe developmental disorder syndrome prevent secretion of CL-K1 from mammalian cells, probably as a result of a failure to bind Ca^2+^ during biosynthesis. The resulting protein deficiency would prevent normal developmental processes mediated via CL-K1/MASP-3 complexes leading to 3MC syndrome.

## Methods

### Proteins

Bovine fetuin and RNAse B, human milk lactoferrin, yeast invertase and yeast mannan were from Sigma-Aldrich Company Ltd. (Gillingham, UK). Bovine thyroglobulin was from GE Healthcare (Little Chalfont, UK). Human IgM and IgG were from Athens Research & Technology, Inc. (Athens, Georgia, USA). Recombinant HIV gp120 was produced in HEK293 cells [27]. Recombinant human MBL was produced in CHO cells as described for rat MBL [28].

#### Production of full-length CL-K1 and trimeric fragments

For full-length protein, the cDNA was cloned into the polylinker region of expression vector pED [29] and protein was produced in Chinese hamster ovary cells and purified by affinity chromatography on mannose-Sepharose columns as described previously for MBL [28,30]. A fragment of the cDNA encoding the neck and CRD (beginning at residue Ser^116^) was cloned into the polylinker of pET28a. Cells were grown in Power Prime broth (Molecular Dimensions Limited (Newmarket, UK)), induced during mid-log phase with IPTG (1 mM), and harvested after growth at 37°C for an additional 16 hours. Inclusion bodies were isolated and resuspended in 50 mM Tris–HCl, containing 8 M urea and 5 mM dithiotreitol (DTT) and protein was refolded by drop dilution into 50 mM Tris–HCl pH 8.0, containing 9.6 mM NaCl, 0.4 mM KCl, 2 mM MgCl_2_, 2 mM CaCl_2_, 0.5 M arginine, 0.05% polyethylene glycol 3,550, 1 mM GSH and 0.1 mM GSSH at a final protein concentration of 0.1 mg/ml. Proteins were purified by ion exchange chromatography on a 10 mL Q-Sepharose column, using a 0.05 to 1 M gradient of NaCl in 20 mM Tris–HCl at pH 8.0, followed by gel filtration on a Superdex 75 16/60 column (GE Healthcare) in 20 mM Tris at pH 7.5 containing 50 mM NaCl and 2 mM CaCl_2_.

#### Binding of CL-K1 to glycoproteins

Two-fold serial dilutions of glycoprotein (0.5 μl at a starting concentration of 2 mg/ml) were spotted on to a nitrocellulose membrane and allowed to dry for 30 minutes at room temperature. The membrane was blocked with 5% milk powder in 25 mM Tris–HCl, pH 7.4 containing 150 mM NaCl for two hours, washed with buffer + 0.5% Tween-20 and then incubated with biotinylated CL-K1 (0.06 mg/ml) at room temperature for one hour. After washing, the membrane was incubated with Streptavidin-ALP conjugate (2ug/ml; Life Technologies Ltd (Paisley, UK)) and developed with BCIP-NBT substrate (Sigma).

#### Glycan-array screen

Human CL-K1 directly labelled with Alexα-488 was diluted to 200 μg/ml in 20 mM Tris–HCl, pH 7.4 containing 150 mM NaCl, 2 mM MgCl_2_, 12 mM CaCl_2_, 1% BSA and 0.05% Tween 20. Labelled protein (70 μL) was applied to the printed surface of the microarray chip coated with natural and synthetic glycans *via* amino linkers. The chip was covered and incubated at room temperature in a humidified chamber for one hour. Binding was detected by measuring the fluorescence associated with each glycan. Data are the average of six replicates.

#### Surface plasmon resonance

CL-K1 was immobilised on the surface of a GLM sensor chip (Bio-Rad Laboratories Ltd (Hemel Hempstead, UK)) by amine coupling. Binding was measured in 10 mM Tris–HCl, pH 7.4 containing 140 mM NaCl, 2 mM CaCl_2_ and 0.005% Tween 20 at a flow rate of 25 μl/minute and at 25°C. The protein surface was regenerated in buffer containing 1 M NaCl and 5 mM EDTA.

#### Complement activation

Complement activation was measured by deposition of complement component C3b on immobilised mannan. Microtiter plates (Nunc) were coated with 2 μg mannan overnight and incubated with 1/100 dilution of whole human serum, previously depleted of endogenous lectins by passage through two mannose-Sepharose columns (2 × 0.5 ml matrix/mL of serum) in 4 mM barbital, 145 mM NaCl, 2 mM CaCl_2_, 1 mM MgCl_2_, pH 7.4, supplemented with recombinant human MBL or human CL-K1. After incubation at 37°C for one hour and washes with buffer containing 0.5% Tween-20, C3b was detected using rabbit anti-human C3c antibody (Dako UK Ltd (Ely, UK)) with alkaline phosphatase-conjugated goat anti-rabbit secondary antibody (Sigma) and p-nitrophenyl phosphate as the substrate.

#### Circular dichroism

All measurements were recorded on a Jasco J-715 spectropolarimeter in 10 mM phosphate buffer, pH 7.4 and at 25°C using 6 μM protein.

#### Fluorescence spectroscopy

All experiments were carried out using a Fluoromax-4 (Horiba Jobin Yvon, Horiba UK Ltd (Middlesex, UK)) instrument with excitation wavelength at 280 nm, slit widths of 2 nm and emission wavelength range of 300 to 400 nm. The λ_ex_ was 280 nm. Protein samples (0.5 μM) in 10 mM Tris–HCl, pH 7.4 containing 140 mM NaCl were measured in the presence of 2 mM CaCl_2_, 1 mM EDTA and 6 M urea. For urea denaturation, proteins (0.5 μM) were equilibrated in urea (0 to 6 M) for 15 minutes at room temperature and measurements were recorded at λ_em_ of 345 nm.

#### Proteolysis

CL-K1 (0.5 mg/ml) was incubated with trypsin (maximum concentration of 4% w/w) in 50 mM Tris–HCl pH 7.5, containing 140 mM NaCl for one hour at 37°C.

#### Binding assays

MaxiSorb microtiter plates (Nunc) were coated with yeast invertase by overnight incubation in 0.2 M carbonate-bicarbonate buffer at pH 9.6 and at 4°C. Wells were blocked with 5% BSA in 10 mM Tris-Cl, 140 mM NaCl, pH 7.4 and incubated with biotinylated CL-K1 (50 nM) mixed with dilutions of carbohydrate ligand for one hour at room temperature. After washing, wells were incubated with 1:2000 dilution of goat peroxidase conjugated anti-biotin antibody (Sigma) and developed with tetramethylbenzidine (Sigma). Absorbance was measured at 450 nM. CL-K1 binding to invertase was carried out in the same way except that plates were coated with CL-K1 and incubated with dilutions of biotinylated invertase.

#### Crystallization and structure determination

All crystals were grown using the sitting-drop vapour diffusion method by mixing equal volumes (1.2 + 1.2 μL) of protein and reservoir solution. Protein at approximately 3 mg/ml was mixed with 10% (vol/vol) ethanol in 100 mM HEPES pH 7.0 containing 4 mM CaCl_2_. Similar conditions were used to crystallize the complex, except that Man(α1-2)Man (1 mM) was included in the mixture. All crystals were transferred to reservoir solution containing 20% (vol/vol) glycerol, before storage in liquid nitrogen and were maintained at 100 K during data collection. Ligand (1 mM) was included in the cryoprotection buffer for the complex. Diffraction data were collected at beamlines I04 and I04-1 at Diamond Light Source and were processed with iMosflm [31]. Phases were determined by molecular replacement with Phaser [32] using the structure of pulmonary-surfactant protein D [33] as a search model (PDB: 1B08). Models were optimized by using cycles of manual refinement with Coot [34] and refinement in Refmac5 [35], part of the CCP4 software suite [36], and in Phenix [37].

## Additional file

Additional file 1:
**Glycan-array analysis of CL-K1 in the presence and absence of Ca** ^**2+**^ **.**

## Abbreviations

3MC: Carnevale, Mingarelli, Malpuech and Michels
BSA: bovine serum albumin
CL-K1: collectin-K1
CRD: carbohydrate-recognition domain
EDTA: ethylenediaminetetraacetic acid
ER: endoplasmic reticulum
MASPs: MBL-associated serine proteases
MBL: mannan-binding lectin
