# Supplementary material for: Molecular basis of sugar recognition by collectin-K1 and the effects of mutations associated with 3MC syndrome
Source: BMC Biol. 2015 Apr 17;13:27. doi: 10.1186/s12915-015-0136-2 (PMC4431178; doi:10.1186/s12915-015-0136-2)
Supplement: Additional file 1: — Glycan-array analysis of CL-K1 in the presence and absence of Ca 2+ . [file 12915_2015_136_MOESM1_ESM.docx]

| **No** | **Ligand** | **With Ca^2+^** | **Without Ca^2+^** |
| --- | --- | --- | --- |
| 1 | Neu5Acα2-8Neu5Acβ-Sp17 | 1305 ± 777 | 8303 ± 4120 |
| 2 | Neu5Acα2-8Neu5Acα2-8Neu5Acβ-Sp8 | 110 ± 46 | 402 ± 138 |
| 3 | Neu5Gcβ2-6Galβ1-4GlcNAc-Sp8 | 930 ± 209 | 4325 ± 514 |
| 4 | Galβ1-3GlcNAcβ1-2Manα1-3(Galβ1-3GlcNAcβ1-2Manα1-6)Manβ1-4GlcNAcβ1-4GlcNAcβ-Sp19 | 9900 ± 1847 | 391 ± 238 |
| 5 | Neu5Acα2-6Galβ1-4GlcNAcβ1-2Manα1-3(Neu5Acα2-6Galβ1-4GlcNAcβ1-2Manα1-6)Manβ1-4GlcNAcβ1-4GlcNAcβ-Sp12 | 115 ± 26 | 810 ± 210 |
| 6 | α-D-Gal–Sp8 | 294 ± 167 | 870 ± 157 |
| 7 | α-D-Glc–Sp8 | 250 ± 80 | 952 ± 297 |
| 8 | α-D-Man–Sp8 | 566 ± 230 | 1156 ± 233 |
| 9 | α-GalNAc–Sp8 | 254 ± 99 | 1607 ± 266 |
| 10 | α-L-Fuc–Sp8 | 431 ± 27 | 1097 ± 138 |
| 11 | α-L-Fuc–Sp9 | 546 ± 98 | 1922 ± 358 |
| 12 | α-L-Rhα-Sp8 | 290 ±158 | 1846 ± 496 |
| 13 | α-Neu5Ac–Sp8 | 714 ± 586 | 3153 ± 596 |
| 14 | α-Neu5Ac–Sp11 | 314 ± 109 | 954 ± 348 |
| 15 | β-Neu5Ac-Sp8 | 670 ± 337 | 4268 ± 2585 |
| 16 | β-D-Gal–Sp8 | 941 ± 308 | 4062 ± 774 |
| 17 | β-D-Glc–Sp8 | 831 ± 505 | 4473 ± 1785 |
| 18 | β-D-Man–Sp8 | 99 ± 9 | 570 ± 170 |
| 19 | β-GalNAc–Sp8 | 206 ± 58 | 1533 ± 69 |
| 20 | β-GlcNAc–Sp0 | 170 ± 91 | 535 ± 116 |
| 21 | β-GlcNAc–Sp8 | 499 ± 293 | 1798 ± 141 |
| 22 | β-GlcN(Gc)-Sp8 | 163 ± 92 | 747 ± 100 |
| 23 | (Galβ1-4GlcNAcb)2-3,6-GalNAcα-Sp8 | 256 ± 45 | 1399 ± 336 |
| 24 | GlcNAcβ1-3(GlcNAcβ1-4)(GlcNAcβ1-6)GlcNAc-Sp8 | 29 ± 62 | 455 ± 105 |
| 25 | [3OSO3][6OSO3]Galβ1-4[6OSO3]GlcNAcβ-Sp0 | 1854 ± 1282 | 6608 ± 1261 |
| 26 | [3OSO3][6OSO3]Galβ1-4GlcNAcβ-Sp0 | 940 ± 438 | 2641 ± 603 |
| 27 | [3OSO3]Galβ1-4Glcβ-Sp8 | 355 ± 62 | 1976 ± 202 |
| 28 | [3OSO3]Galβ1-4(6OSO3)Glcβ–Sp0 | 1537 ± 1082 | 7953 ± 2598 |
| 29 | [3OSO3]Galβ1-4(6OSO3)Glcβ–Sp8 | 271 ± 78 | 2449 ± 1024 |
| 30 | [3OSO3]Galβ1-3(Fucα1-4)GlcNAcβ–Sp8 | 380 ± 115 | 938 ± 139 |
| 31 | [3OSO3]Galβ1-3GalNAcα-Sp8 | 153 ± 11 | 822 ± 78 |
| 32 | [3OSO3]Galβ1-3GlcNAcβ–Sp8 | 409 ± 180 | 2523 ± 418 |
| 33 | [3OSO3]Galβ1-4(Fucα1-3)GlcNAcβ–Sp8 | 1064 ± 459 | 2812 ± 370 |
| 34 | [3OSO3]Galβ1-4[6OSO3]GlcNAcβ-Sp8 | 1337 ± 1017 | 3999 ± 444 |
| 35 | [3OSO3]Galβ1-4GlcNAcβ–Sp0 | 310 ± 172 | 1130 ± 583 |
| 36 | [3OSO3]Galβ1-4GlcNAcβ-Sp8 | 50 ± 46 | 105 ±128 |
| 37 | [3OSO3]Galβ–Sp8 | 57 ± 36 | 15 ± 29 |
| 38 | [4OSO3][6OSO3]Galβ1-4GlcNAcβ-Sp0 | 1527 ± 1213 | 9962 ± 7261 |
| 39 | [4OSO3]Galβ1-4GlcNAcβ-Sp8 | 175 ± 10 | 1160 ± 180 |
| 40 | 6-H2PO3Manα-Sp8 | 262 ± 88 | 1219 ± 303 |
| 41 | [6OSO3]Galβ1-4Glcβ–Sp0 | 267 ± 103 | 1046 ± 397 |
| 42 | [6OSO3]Galβ1-4Glcβ–Sp8 | 447 ± 157 | 1907 ± 207 |
| 43 | [6OSO3]Galβ1-4GlcNAcβ–Sp8 | 84 ± 14 | 999 ± 290 |
| 44 | [6OSO3]Galβ1-4[6OSO3]Glcβ-Sp8 | 724 ± 204 | 4107 ± 436 |
| 45 | NeuAcα2-3[6OSO3]Galβ1-4GlcNAcβ–Sp8 | 338 ± 168 | 1128 ± 303 |
| 46 | [6OSO3]GlcNAcβ–Sp8 | 534 ± 144 | 2971 ± 166 |
| 47 | 9NAcNeu5Acα-Sp8 | 24736 ±1429 | 27736 ±1220 |
| 48 | 9NAcNeu5Acα2-6Galβ1-4GlcNAcβ-Sp8 | 425 ± 248 | 3145 ± 1621 |
| 49 | Manα1-3(Manα1-6)Manβ1-4GlcNAcβ1-4GlcNAcβ-Sp13 | 658 146 | 2274 ± 296 |
| 50 | GlcNAcβ1-2Manα1-3(GlcNAcβ1-2Manα1-6)Manβ1-4GlcNAcβ1-4GlcNAcβ-Sp13 | 880 ± 129 | 4389 ± 1640 |
| 51 | Galβ1-4GlcNAcβ1-2Manα1-3(Galβ1-4GlcNAcβ1-2Manα1-6)Manβ1-4GlcNAcβ1-4GlcNAcβ-Sp13 | 135 ± 57 | 522 ± 277 |
| 52 | Neu5Acα2-6Galβ1-4GlcNAcβ1-2Manα1-3(Neu5Acα2-6Galβ1-4GlcNAcβ1-2Manα1-6)Manβ1-4GlcNAcβ1-4GlcNAcβ-Sp13 | 153 72 | 164 101 |
| 53 | Neu5Acα2-6Galβ1-4GlcNAcβ1-2Manα1-3(Neu5Acα2-6Galβ1-4GlcNAcβ1-2Manα1-6)Manβ1-4GlcNAcβ1-4GlcNAcβ-Sp8 | 127 ± 73 | 402 ± 146 |
| 54 | Fucα1-2Galβ1-3GalNAcβ1-3Galα-Sp9 | 482 ± 231 | 2793 ± 171 |
| 55 | Fucα1-2Galβ1-3GalNAcβ1-3Galα1-4Galβ1-4Glcβ-Sp9 | 73 ± 43 | 507 ± 196 |
| 56 | Fucα1-2Galβ1-3(Fucα1-4)GlcNAcβ–Sp8 | 215 ± 110 | 918 ± 201 |
| 57 | Fucα1-2Galβ1-3GalNAcα-Sp8 | 158 ± 51 | 1010 ± 195 |
| 58 | Fucα1-2Galβ1-3GalNAcβ1-4(Neu5Acα2-3)Galβ1-4Glcβ-Sp0 | 1535 ± 394 | 787 ± 85 |
| 59 | Fucα1-2Galβ1-3GalNAcβ1-4(Neu5Acα2-3)Galβ1-4Glcβ-Sp9 | 792 ± 199 | 3964 ± 404 |
| 60 | Fucα1-2Galβ1-3GlcNAcβ1-3Galβ1-4Glcβ–Sp10 | 154 ± 40 | 1060 ± 132 |
| 61 | Fucα1-2Galβ1-3GlcNAcβ1-3Galβ1-4Glcβ–Sp8 | 211 ± 43 | 785 ± 162 |
| 62 | Fucα1-2Galβ1-3GlcNAcβ–Sp0 | 217 ± 110 | 1274 ± 345 |
| 63 | Fucα1-2Galβ1-3GlcNAcβ–Sp8 | 129 ± 55 | 969 ± 200 |
| 64 | Fucα1-2Galβ1-4(Fucα1-3)GlcNAcβ1-3Galβ1-4(Fucα1-3)GlcNAcβ-Sp0 | 614 ± 447 | 254 ± 52 |
| 65 | Fucα1-2Galβ1-4(Fucα1-3)GlcNAcβ1-3Galβ1-4(Fucα1-3)GlcNAcβ1-3Galβ1-4(Fucα1-3)GlcNAcβ-Sp0 | 6087 ± 1245 | 899 ± 223 |
| 66 | Fucα1-2Galβ1-4(Fucα1-3)GlcNAcβ–Sp0 | 153 ± 64 | 551 ± 102 |
| 67 | Fucα1-2Galβ1-4(Fucα1-3)GlcNAcβ–Sp8 | 6865 ± 4127 | 5434 ± 867 |
| 68 | Fucα1-2Galβ1-4GlcNAcβ1-3Galβ1-4GlcNAc–Sp0 | 171 ± 70 | 763 ± 142 |
| 69 | Fucα1-2Galβ1-4GlcNAcβ1-3Galβ1-4GlcNAcβ1-3Galβ1-4GlcNAcβ-Sp0 | 531 ± 294 | 4480 ± 1440 |
| 70 | Fucα1-2Galβ1-4GlcNAcβ–Sp0 | 382 ± 36 | 2151 ± 360 |
| 71 | Fucα1-2Galβ1-4GlcNAcβ–Sp8 | 411 ± 184 | 2669 ± 665 |
| 72 | Fucα1-2Galβ1-4Glcβ–Sp0 | 145 ± 32 | 800 ± 345 |
| 73 | Fucα1-2Galβ–Sp8 | 195 ± 95 | 511 ± 315 |
| 74 | Fucα1-3GlcNAcβ-Sp8 | 118 ± 57 | 315 ± 79 |
| 75 | Fucα1-4GlcNAcβ–Sp8 | 165 ± 22 | 1397 ± 361 |
| 76 | Fucβ1-3GlcNAcβ-Sp8 | 154 ± 26 | 439 ± 237 |
| 77 | GalNAcα1-3(Fucα1-2)Galβ1-3GlcNAcβ-Sp0 | 1321 ± 417 | 2035 ± 334 |
| 78 | GalNAcα1-3(Fucα1-2)Galβ1-4(Fucα1-3)GlcNAcβ-Sp0 | 577 ± 343 | 1145 ± 335 |
| 79 | GalNAcα1-3(Fucα1-2)Galβ1-4GlcNAcβ-Sp0 | 300 ± 177 | 790 ± 308 |
| 80 | GalNAcα1-3(Fucα1-2)Galβ1-4GlcNAcβ–Sp8 | 294 ± 254 | 1786 ± 1820 |
| 81 | GalNAcα1-3(Fucα1-2)Galβ1-4Glcβ-Sp0 | 479 ± 153 | 899 ± 308 |
| 82 | GalNAcα1-3(Fucα1-2)Galβ–Sp8 | 266 ± 97 | 756 ± 200 |
| 83 | GalNAcα1-3GalNAcβ–Sp8 | 192 ± 55 | 1214 ± 177 |
| 84 | GalNAcα1-3Galβ–Sp8 | 430 ± 153 | 2395 ± 461 |
| 85 | GalNAcα1-4(Fucα1-2)Galβ1-4GlcNAcβ-Sp8 | 114 ± 33 | 253 ± 61 |
| 86 | GalNAcβ1-3GalNAcα-Sp8 | 160 ± 45 | 737 ± 160 |
| 87 | GalNAcβ1-3(Fucα1-2)Galβ-Sp8 | 85 ± 53 | 387 ± 169 |
| 88 | GalNAcβ1-3Galα1-4Galβ1-4GlcNAcβ-Sp0 | 2388 ± 1406 | 834 ± 151 |
| 89 | GalNAcβ1-4(Fucα1-3)GlcNAcβ-Sp0 | 1381 ± 524 | 1088 ± 398 |
| 90 | GalNAcβ1-4GlcNAcβ–Sp0 | 154 ± 73 | 1124 ± 306 |
| 91 | GalNAcβ1-4GlcNAcβ–Sp8 | 146 ± 34 | 841 ± 210 |
| 92 | Galα1-2Galβ–Sp8 | 385 ± 276 | 2676 ± 990 |
| 93 | Galα1-3(Fucα1-2)Galβ1-3GlcNAcβ-Sp0 | 293 ± 230 | 899 ± 295 |
| 94 | Galα1-3(Fucα1-2)Galβ1-4(Fucα1-3)GlcNAcβ-Sp0 | 683 ± 269 | 3865 ± 423 |
| 95 | Galα1-3(Fucα1-2)Galβ1-4GlcNAc-Sp0 | 234 ± 59 | 1574 ± 411 |
| 96 | Galα1-3(Fucα1-2)Galβ1-4Glcβ-Sp0 | 421 ± 98 | 2604 ± 585 |
| 97 | Galα1-3(Fucα1-2)Galβ–Sp8 | 176 ± 46 | 1039 ± 386 |
| 98 | Galα1-3(Galα1-4)Galβ1-4GlcNAcβ-Sp8 | 194 ± 114 | 500 ± 252 |
| 99 | Galα1-3GalNAcα-Sp8 | 91 ± 43 | 425 ± 143 |
| 100 | Galα1-3GalNAcβ–Sp8 | 292 ± 171 | 1181 ± 380 |
| 101 | Galα1-3Galβ1-4(Fucα1-3)GlcNAcβ–Sp8 | 170 ± 77 | 1159 ± 159 |
| 102 | Galα1-3Galβ1-3GlcNAcβ-Sp0 | 281 ± 152 | 846 ± 190 |
| 103 | Galα1-3Galβ1-4GlcNAcβ–Sp8 | 530 ± 192 | 2977 ± 911 |
| 104 | Galα1-3Galβ1-4Glcβ–Sp0 | 120 ± 50 | 508 ± 71 |
| 105 | Galα1-3Galβ–Sp8 | 639 ± 114 | 4532 ± 1150 |
| 106 | Galα1-4(Fucα1-2)Galβ1-4GlcNAcβ-Sp8 | 136 ± 32 | 961 ± 156 |
| 107 | Galα1-4Galβ1-4GlcNAcβ–Sp0 | 1030 ± 337 | 4653 ± 1392 |
| 108 | Galα1-4Galβ1-4GlcNAcβ–Sp8 | 128 ± 23 | 849 ± 227 |
| 109 | Galα1-4Galβ1-4Glcβ–Sp0 | 118 ± 30 | 468 ± 124 |
| 110 | Galα1-4GlcNAcβ–Sp8 | 179 ± 161 | 662 ± 282 |
| 111 | Galα1-6Glcβ-Sp8 | 192 ± 55 | 1603 ± 207 |
| 112 | Galβ1-2Galβ–Sp8 | 307 ± 175 | 944 ± 246 |
| 113 | Galβ1-3(Fucα1-4)GlcNAcβ1-3Galβ1-4(Fucα1-3)GlcNAcβ-Sp0 | 686 ± 925 | 886 ± 322 |
| 114 | Galβ1-3(Fucα1-4)GlcNAcβ1-3Galβ1-4GlcNAcβ-Sp0 | 1451 ± 1015 | 563 ± 138 |
| 115 | Galβ1-3(Fucα1-4)GlcNAc–Sp0 | 459 ± 356 | 4012 ± 991 |
| 116 | Galβ1-3(Fucα1-4)GlcNAc–Sp8 | 287 ± 84 | 1294 ± 686 |
| 117 | Galβ1-3(Fucα1-4)GlcNAcβ–Sp8 | 509 ± 198 | 3708 ± 451 |
| 118 | Galβ1-3(Galβ1-4GlcNAcβ1-6)GalNAcα-Sp8 | 258 ± 104 | 513 ± 82 |
| 119 | Galβ1-3(GlcNAcβ1-6)GalNAcα-Sp8 | 360 ± 134 | 2777 ± 815 |
| 120 | Galβ1-3(Neu5Acα2-6)GalNAcα-Sp8 | 128 ± 45 | 585 ± 128 |
| 121 | Galβ1-3(Neu5Acβ2-6)GalNAcα-Sp8 | 285 ± 96 | 1730 ± 332 |
| 122 | Galβ1-3(Neu5Acα2-6)GlcNAcβ1-4Galβ1-4Glcβ-Sp10 | 249 ± 108 | 628 ± 285 |
| 123 | Galβ1-3GalNAcα-Sp8 | 171 ± 39 | 1672 ± 317 |
| 124 | Galβ1-3GalNAcβ–Sp8 | 215 ± 109 | 918 ± 102 |
| 125 | Galβ1-3GalNAcβ1-3Galα1-4Galβ1-4Glcβ-Sp0 | 558 ± 369 | 764 ± 244 |
| 126 | Galβ1-3GalNAcβ1-4(Neu5Acα2-3)Galβ1-4Glcβ-Sp0 | 802 ± 282 | 3671 ± 1581 |
| 127 | Galβ1-3GalNAcβ1-4Galβ1-4Glcβ–Sp8 | 219 ± 93 | 905 ± 320 |
| 128 | Galβ1-3Galβ–Sp8 | 866 ± 494 | 3855 ± 1724 |
| 129 | Galβ1-3GlcNAcβ1-3Galβ1-4GlcNAcβ-Sp0 | 367 ± 192 | 577 ± 120 |
| 130 | Galβ1-3GlcNAcβ1-3Galβ1-4Glcβ–Sp10 | 475 ± 91 | 3320 ± 470 |
| 131 | Galβ1-3GlcNAcβ–Sp0 | 195 ± 43 | 1314 ± 235 |
| 132 | Galβ1-3GlcNAcβ–Sp8 | 593 ± 185 | 2470 ± 576 |
| 133 | Galβ1-4(Fucα1-3)GlcNAcβ–Sp0 | 187 ± 40 | 445 ± 101 |
| 134 | Galβ1-4(Fucα1-3)GlcNAcβ–Sp8 | 265 ± 131 | 1170 ± 431 |
| 135 | Galβ1-4(Fucα1-3)GlcNAcβ1-4Galβ1-4(Fucα1-3)GlcNAcβ-Sp0 | 1505 ± 856 | 443 ± 196 |
| 136 | Galβ1-4(Fucα1-3)GlcNAcβ1-4Galβ1-4(Fucα1-3)GlcNAcβ1-4Galβ1-4(Fucα1-3)GlcNAcβ–Sp0 | 258 ± 39 | 975 ± 56 |
| 137 | Galβ1-4[6OSO3]Glcβ–Sp0 | 295 ± 153 | 935 ± 194 |
| 138 | Galβ1-4[6OSO3]Glcβ–Sp8 | 353 ± 88 | 3313 v1840 |
| 139 | Galβ1-4GalNAcα1-3(Fucα1-2)Galβ1-4GlcNAcβ-Sp8 | 150 ± 75 | 476 ± 84 |
| 140 | Galβ1-4GalNAcβ1-3(Fucα1-2)Galβ1-4GlcNAcβ-Sp8 | 804 ± 1145 | 3120 ± 103 |
| 141 | Neu5Acα2-3Galβ1-4GlcNAcβ1-2Manα1-3(Neu5Acα2-3Galβ1-4GlcNAcβ1-2Manα1-6)Manβ1-4GlcNAcβ1-4GlcNAcβ-Sp12 | 53 ± 15 | 353 ± 153 |
| 142 | Galβ1-4GlcNAcβ1-3GalNAcα-Sp8 | 161 ± 29 | 650 ± 201 |
| 143 | Galβ1-4GlcNAcβ1-3Galβ1-4(Fucα1-3)GlcNAcβ1-3Galβ1-4(Fucα1-3)GlcNAcβ-Sp0 | 736 ± 175 | 456 ± 127 |
| 144 | Galβ1-4GlcNAcβ1-3Galβ1-4GlcNAcβ1-3Galβ1-4GlcNAcβ–Sp0 | 501 ± 203 | 2281 ± 832 |
| 145 | Galβ1-4GlcNAcβ1-3Galβ1-4GlcNAcβ–Sp0 | 141 ± 16 | 513 ± 180 |
| 146 | Galβ1-4GlcNAcβ1-3Galβ1-4Glcβ–Sp0 | 281 ± 190 | 1026 ± 154 |
| 147 | Galβ1-4GlcNAcβ1-3Galβ1-4Glcβ–Sp8 | 104 ± 36 | 407 ± 98 |
| 148 | Galβ1-4GlcNAcβ1-6(Galβ1-3)GalNAcα-Sp8 | 437 ± 328 | 1043 ± 233 |
| 149 | Galβ1-4GlcNAcβ1-6GalNAcα-Sp8 | 157 ± 78 | 583 ± 113 |
| 150 | Galβ1-4GlcNAcβ–Sp0 | 149 ± 71 | 1182 ± 324 |
| 151 | Galβ1-4GlcNAcβ–Sp8 | 196 ± 109 | 1721 ± 705 |
| 152 | Galβ1-4Glcβ–Sp0 | 123 ± 52 | 1038 ± 227 |
| 153 | Galβ1-4Glcβ–Sp8 | 532 ± 340 | 4102 ± 637 |
| 154 | GlcNAcα1-3Galβ1-4GlcNAcβ-Sp8 | 131 ± 55 | 589 ± 183 |
| 155 | GlcNAcα1-6Galβ1-4GlcNAcβ-Sp8 | 367 ± 236 | 2116 ± 726 |
| 156 | GlcNAcβ1-2Galβ1-3GalNAcα-Sp8 | 129 ± 39 | 596 ± 262 |
| 157 | GlcNAcβ1-3(GlcNAcβ1-6)GalNAcα-Sp8 | 114 ± 46 | 699 ± 77 |
| 158 | GlcNAcβ1-3(GlcNAcβ1-6)Galβ1-4GlcNAcβ–Sp8 | 157 ± 72 | 352 ± 110 |
| 159 | GlcNAcβ1-3GalNAcα-Sp8 | 129 ± 32 | 881 ± 70 |
| 160 | GlcNAcβ1-3Galβ-Sp8 | 137 ± 106 | 463 ± 143 |
| 161 | GlcNAcβ1-3Galβ1-3GalNAcα-Sp8 | 473 ± 94 | 2617 ± 947 |
| 162 | GlcNAcβ1-3Galβ1-4GlcNAcβ–Sp0 | 218 ± 27 | 1394 ± 1031 |
| 163 | GlcNAcβ1-3Galβ1-4GlcNAcβ-Sp8 | 136 ± 42 | 326 ± 196 |
| 164 | GlcNAcβ1-3Galβ1-4GlcNAcβ1-3Galβ1-4GlcNAcβ-Sp0 | 247 ± 21 | 1139 ± 566 |
| 165 | GlcNAcβ1-3Galβ1-4Glcβ–Sp0 | 255 ± 32 | 669 ± 71 |
| 166 | GlcNAcβ1-4MDPLys | 549 ± 398 | 4117 ± 1089 |
| 167 | GlcNAcβ1-4(GlcNAcβ1-6)GalNAcα-Sp8 | 158 ± 25 | 661 ± 110 |
| 168 | GlcNAcβ1-4Galβ1-4GlcNAcβ-Sp8 | 276 ± 62 | 2112 ± 1182 |
| 169 | (GlcNAcβ1-4)6β-Sp8 | 71 ± 33 | 347 ± 140 |
| 170 | (GlcNAcβ1-4)5β-Sp8 | 114 ± 30 | 696 ± 167 |
| 171 | GlcNAcβ1-4GlcNAcβ1-4GlcNAcβ–Sp8 | 154 ± 21 | 553 ± 430 |
| 172 | GlcNAcβ1-6(Galβ1-3)GalNAcα-Sp8 | 149 ± 117 | 692 ± 176 |
| 173 | GlcNAcβ1-6GalNAcα-Sp8 | 213 ± 108 | 368 ± 74 |
| 174 | GlcNAcβ1-6Galβ1-4GlcNAcβ-Sp8 | 234 ± 61 | 1209 ± 363 |
| 175 | Glcα1-4Glcβ–Sp8 | 103 ± 22 | 641 ± 75 |
| 176 | Glcα1-4Glcα-Sp8 | 398 ± 477 | 2041 ± 342 |
| 177 | Glcα1-6Glcα1-6Glcβ-Sp8 | 360 ± 189 | 1040 ± 513 |
| 178 | Glcβ1-4Glcβ-Sp8 | 234 ± 166 | 2546 ± 1126 |
| 179 | Glcβ1-6Glcβ-Sp8 | 181 ± 86 | 992 ± 293 |
| 180 | G-ol-Sp8 | 591 ± 289 | 3538 ± 2387 |
| 181 | GlcAα-Sp8 | 140 ± 28 | 630 ± 261 |
| 182 | GlcAβ-Sp8 | 148 ± 17 | 1009 ± 471 |
| 183 | GlcAβ1-3Galβ-Sp8 | 193 ± 154 | 634 ± 227 |
| 184 | GlcAβ1-6Galβ-Sp8 | 316 ± 197 | 1899 ± 297 |
| 185 | KDNα2-3Galβ1-3GlcNAcβ–Sp0 | 140 ± 44 | 322 ± 44 |
| 186 | KDNα2-3Galβ1-4GlcNAcβ–Sp0 | 258 ± 120 | 1966 ± 316 |
| 187 | Manα1-2Manα1-2Manα1-3Manα-Sp9 | 27836 ± 793 | 127 ± 35 |
| 188 | Manα1-2Manα1-3(Manα1-2Manα1-6)Manα-Sp9 | 32562 ± 238 | 1023 ± 105 |
| 189 | Manα1-2Manα1-3Manα-Sp9 | 20778 ± 1634 | 615 ± 161 |
| 190 | Manα1-6(Manα1-2Manα1-3)Manα1-6(Manα2Manα1-3)Manβ1-4GlcNAcβ1-4GlcNAcβ-Sp12 | 30144 ± 459 | 2489 ± 1258 |
| 191 | Manα1-2Manα1-6(Manα1-3)Manα1-6(Manα2Manα2Manα1-3)Manβ1-4GlcNAcβ1-4GlcNAcβ-Sp12 | 31688 ± 1754 | 894 ± 89 |
| 192 | Manα1-2Manα1-2Manα1-3(Manα1-2Manα1-3(Manα1-2Manα1-6)Manα1-6)Manβ1-4GlcNAcβ1-4GlcNAcβ-Sp12 | 23515 ± 1853 | 3497 ± 1096 |
| 193 | Manα1-3(Manα1-6)Manα-Sp9 | 134 ± 50 | 570 ± 308 |
| 194 | Manα1-3(Manα1-2Manα1-2Manα1-6)Manα-Sp9 | 29239 ± 988 | 455 ± 65 |
| 195 | Manα1-6(Manα1-3)Manα1-6(Manα2Manα1-3)Manβ1-4GlcNAcβ1-4GlcNAcβ-Sp12 | 27447 ± 1106 | 555 ± 239 |
| 196 | Manα1-6(Manα1-3)Manα1-6(Manα1-3)Manβ1-4GlcNAcβ1-4GlcNAcβ-Sp12 | 1729 ± 498 | 871 ± 241 |
| 197 | Neu5Acα2-6Galβ1-4GlcNAcβ1-2Manα1-3(Neu5Acα2-3Galβ1-4GlcNAcβ1-2Manα1-6)Manβ1-4GlcNAcβ1-4GlcNAcβ-Sp12 | 188 ± 116 | 181± 64 |
| 198 | Manβ1-4GlcNAcβ-Sp0 | 123 ± 145 | 507 ± 22 |
| 199 | Fucα1-3(Galβ1-4)GlcNAcβ1-2Manα1-3(Fucα1-3(Galβ1-4)GlcNAcβ1-2Manα1-6)Manβ1-4GlcNAcβ1-4GlcNAcβ-Sp20 | 128 ± 63 | 1111 ± 41 |
| 200 | Neu5Acα2-3Galβ1-3GalNAcα-Sp8 | 115 ± 53 | 362 ± 120 |
| 201 | NeuAcα2-8NeuAcα2-8NeuAcα2-8NeuAcα2-3(GalNAcβ1-4)Galβ1-4Glcβ-Sp0 | 6073 ± 4649 | 9538 ± 7611 |
| 202 | Neu5Acα2-8Neu5Acα2-8Neu5Acα2-3(GalNAcβ1-4)Galβ1-4Glcβ-Sp0 | 983 ± 417 | 938 ± 173 |
| 203 | Neu5Acα2-8Neu5Acα2-8Neu5Acα2-3Galβ1-4Glcβ–Sp0 | 122 ± 37 | 655 ± 67 |
| 204 | Neu5Acα2-8Neu5Acα2-3(GalNAcβ1-4)Galβ1-4Glcβ–Sp0 | 3129 ± 480 | 400 ± 229 |
| 205 | Neu5Acα2-8Neu5Acα2-8Neu5Acα-Sp8 | 258 ± 39 | 1442 ± 121 |
| 206 | Neu5Acα2-3(6-O-Su)Galβ1-4(Fucα1-3)GlcNAcβ–Sp8 | 111 ± 54 | 503 ± 209 |
| 207 | Neu5Acα2-3(GalNAcβ1-4)Galβ1-4GlcNAcβ-Sp0 | 560 ± 189 | 1493 ± 207 |
| 208 | Neu5Acα2-3(GalNAcβ1-4)Galβ1-4GlcNAcβ-Sp8 | 6539 ± 2809 | 346 ± 117 |
| 209 | Neu5Acα2-3(GalNAcβ1-4)Galβ1-4Glcβ–Sp0 | 225 ± 46 | 1608 ± 301 |
| 210 | NeuAcα2-3(NeuAcα2-3Galβ1-3GalNAcβ1-4)Galβ1-4Glcβ-Sp0 | 1577 ± 812 | 302 ± 45 |
| 211 | Neu5Acα2-3(Neu5Acα2-6)GalNAcα-Sp8 | 655 ± 497 | 4100 ± 3592 |
| 212 | Neu5Acα2-3GalNAcα-Sp8 | 201 ± 36 | 843 ± 91 |
| 213 | Neu5Acα2-3GalNAcβ1-4GlcNAcβ-Sp0 | 315 ± 223 | 3457 ± 2263 |
| 214 | Neu5Acα2-3Galβ1-3(6OSO3)GlcNAc-Sp8 | 240 ± 32 | 1800 ± 237 |
| 215 | Neu5Acα2-3Galβ1-3(Fucα1-4)GlcNAcβ–Sp8 | 390 ± 319 | 4566 ± 577 |
| 216 | NeuAcα2-3Galβ1-3(Fucα1-4)GlcNAcβ1-3Galβ1-4(Fucα1-3)GlcNAcb Sp0 | 4910 ± 559 | 524 ± 104 |
| 217 | Neu5Acα2-3Galβ1-3(Neu5Acα2-3Galβ1-4)GlcNAcβ-Sp8 | 958 ± 288 | 6006 ± 1414 |
| 218 | Neu5Acα2-3Galβ1-3[6OSO3]GalNAcα-Sp8 | 197 ± 75 | 1964 ± 454 |
| 219 | Neu5Acα2-3Galβ1-3(Neu5Acα2-6)GalNAcα-Sp8 | 113 ± 26 | 599 ± 210 |
| 220 | Neu5Acα2-3Galβ-Sp8 | 156 ± 47 | 872 ± 309 |
| 221 | NeuAcα2-3Galβ1-3GalNAcβ1-3Galα1-4Galβ1-4Glcβ-Sp0 | 3802 ± 1448 | 779 ± 135 |
| 222 | NeuAcα2-3Galβ1-3GlcNAcβ1-3Galβ1-4GlcNAcβ-Sp0 | 589 ± 200 | 257 ± 184 |
| 223 | Neu5Acα2-3Galβ1-3GlcNAcβ–Sp0 | 362 ± 105 | 696 ± 235 |
| 224 | Neu5Acα2-3Galβ1-3GlcNAcβ–Sp8 | 326 ± 69 | 1850 ± 272 |
| 225 | Neu5Acα2-3Galβ1-4[6OSO3]GlcNAcβ-Sp8 | 201 ± 23 | 949 ± 109 |
| 226 | Neu5Acα2-3Galβ1-4(Fucα1-3)(6OSO3)GlcNAcβ–Sp8 | 1720 ± 967 | 12797 ± 5349 |
| 227 | Neu5Acα2-3Galβ1-4(Fucα1-3)GlcNAcβ1-3Galβ1-4(Fucα1-3)GlcNAcβ1-3Galβ1-4(Fucα1-3)GlcNAcβ–Sp0 | 1113 ± 226 | 255 ± 68 |
| 228 | Neu5Acα2-3Galβ1-4(Fucα1-3)GlcNAcβ–Sp0 | 582 ± 141 | 3579 ± 463 |
| 229 | Neu5Acα2-3Galβ1-4(Fucα1-3)GlcNAcβ–Sp8 | 84 ± 30 | 412 ± 227 |
| 230 | Neu5Acα2-3Galβ1-4(Fucα1-3)GlcNAcβ1-3Galβ-Sp8 | 79 ± 32 | 365 ± 204 |
| 231 | Neu5Acα2-3Galβ1-4(Fucα1-3)GlcNAcβ1-3Galβ1-4GlcNAcβ-Sp8 | 2081 ± 478 | 448 ± 146 |
| 232 | Neu5Acα2-3Galβ1-4GlcNAcβ1-3Galβ1-4(Fucα1-3)GlcNAc-Sp0 | 2188 ± 625 | 3190 ± 249 |
| 233 | Neu5Acα2-3Galβ1-4GlcNAcβ1-3Galβ1-4GlcNAcβ1-3Galβ1-4GlcNAcβ–Sp0 | 156 ± 56 | 498 ± 47 |
| 234 | Neu5Acα2-3Galβ1-4GlcNAcβ–Sp0 | 152 ± 52 | 330 ± 79 |
| 235 | Neu5Acα2-3Galβ1-4GlcNAcβ–Sp8 | 226 ± 58 | 1554 ± 293 |
| 236 | Neu5Acα2-3Galβ1-4GlcNAcβ1-3Galβ1-4GlcNAcβ-Sp0 | 208 ± 198 | 278 ± 32 |
| 237 | Neu5Acα2-3Galβ1-4Glcβ–Sp0 | 250 ± 126 | 1011 ± 186 |
| 238 | Neu5Acα2-3Galβ1-4Glcβ–Sp8 | 685 ± 335 | 4510 ± 246 |
| 239 | Galβ1-4GlcNAcβ1-2Manα1-3(Fucα1-3(Galβ1-4)GlcNAcβ1-2Manα1-6)Manβ1-4GlcNAcβ1-4GlcNAcβ-Sp20 | 91 ± 62 | 45 ± 21 |
| 240 | Neu5Acα2-6GalNAcα-Sp8 | 148 ± 51 | 703 ± 141 |
| 241 | Neu5Acα2-6GalNAcβ1-4GlcNAcβ-Sp0 | 518 ± 159 | 2390 ± 407 |
| 242 | Neu5Acα2-6Galβ1-4[6OSO3]GlcNAcβ-Sp8 | 114 ± 74 | 1229 ± 710 |
| 243 | Neu5Acα2-6Galβ1-4GlcNAcβ–Sp0 | 131 ± 46 | 641 ± 340 |
| 244 | Neu5Acα2-6Galβ1-4GlcNAcβ–Sp8 | 215 ± 41 | 645 ± 74 |
| 245 | Neu5Acα2-6Galβ1-4GlcNAcβ1-3Galβ1-4(Fucα1-3)GlcNAcβ1-3Galβ1-4(Fucα1-3)GlcNAcβ-Sp0 | 11168 ± 2814 | 677 ± 97 |
| 246 | Neu5Acα2-6Galβ1-4GlcNAcβ1-3Galβ1-4GlcNAcβ-Sp0 | 117 ± 35 | 183 ± 62 |
| 247 | Neu5Acα2-6Galβ1-4Glcβ–Sp0 | 318 ± 91 | 1740 ± 242 |
| 248 | Neu5Acα2-6Galβ1-4Glcβ–Sp8 | 216 ± 110 | 939 ± 353 |
| 249 | Neu5Acα2-6Galβ–Sp8 | 552 ± 337 | 2257 ± 365 |
| 250 | Neu5Acα2-8Neu5Acα-Sp8 | 91 ± 85 | 830 ± 202 |
| 251 | Neu5Acα2-8Neu5Acα2-3Galβ1-4Glcβ–Sp0 | 846 ± 492 | 7003 ± 2487 |
| 252 | Neu5Acβ2-6GalNAcα-Sp8 | 749 ± 417 | 3711 ± 1426 |
| 253 | Neu5Acβ2-6Galβ1-4GlcNAcβ-Sp8 | 93 ± 36 | 447 ± 313 |
| 254 | Galβ1-4GlcNAcβ1-2Manα1-3(Neu5Acα2-6Galβ1-4GlcNAcβ1-2Manα1-6)Manβ1-4GlcNAcβ1-4GlcNAcβ-Sp21 | 569 ± 335 | 4957 ± 1440 |
| 255 | Neu5Gcα2-3Galβ1-3(Fucα1-4)GlcNAcβ-Sp0 | 581 ± 143 | 789 ± 265 |
| 256 | Neu5Gcα2-3Galβ1-3GlcNAcβ-Sp0 | 173 ± 129 | 354 ± 166 |
| 257 | Neu5Gcα2-3Galβ1-4(Fucα1-3)GlcNAcβ-Sp0 | 733 ± 354 | 954 ± 170 |
| 258 | Neu5Gcα2-3Galβ1-4GlcNAcβ–Sp0 | 171 ± 53 | 343 ± 112 |
| 259 | Neu5Gcα2-3Galβ1-4Glcβ–Sp0 | 321 ± 92 | 1965 ± 185 |
| 260 | Neu5Gcα2-6GalNAcα-Sp0 | 233 ± 46 | 1431 ± 508 |
| 261 | Neu5Gcα2-6Galβ1-4GlcNAcβ–Sp0 | 519 ± 196 | 2435 ± 50 |
| 262 | Neu5Gcα-Sp8 | 460 ± 278 | 1955 ± 543 |
| 263 | [3OSO3]Galβ1-4(Fucα1-3)(6OSO3)Glc-Sp0 | 1498 ± 1119 | 7102 ± 3847 |
| 264 | [3OSO3]Galβ1-4(Fucα1-3)Glc-Sp0 | 1098 ± 422 | 2710 ± 179 |
| 265 | [3OSO3]Galβ1-4[Fucα1-3][6OSO3]GlcNAc-Sp8 | 195 ± 80 | 769 ± 260 |
| 266 | [3OSO3]Galβ1-4[Fucα1-3]GlcNAc-Sp0 | 182 ± 89 | 710 ± 266 |
| 267 | Fucα1-2[6OSO3]Galβ1-4GlcNAc-Sp0 | 577 ± 267 | 2565 ± 485 |
| 268 | Fucα1-2Galβ1-4[6OSO3]GlcNAc-Sp8 | 204 ± 129 | 707 ± 126 |
| 269 | Fucα1-2[6OSO3]Galβ1-4[6OSO3]Glc-Sp0 | 1260 ± 677 | 6403 ± 4273 |
| 270 | Fucα1-2-(6OSO3)-Galβ1-4Glc-Sp0 | 618 ± 195 | 2921 ± 267 |
| 271 | Fucα1-2-Galβ1-4[6OSO3]Glc-Sp0 | 586 ± 243 | 2525 ± 193 |
| 272 | Galβ1-3(Fucα1-4)GlcNAcβ1-3Galβ1-3(Fucα1-4)GlcNAcβ-Sp0 | 1290 ± 313 | 608 ± 96 |
| 273 | Galβ1-3-(Galβ1-4GlcNacβ1-6)GalNAc-Sp14 | 346 ± 186 | 2121 ± 839 |
| 274 | Galβ1-3(GlcNacβ1-6)GalNAc-Sp14 | 687 ± 377 | 749 ± 241 |
| 275 | Galβ1-3-(Neu5Aα2-3Galβ1-4GlcNacβ1-6)GalNAc-Sp14 | 424 ± 312 | 1995 ± 831 |
| 276 | Galβ1-3GalNAc-Sp14 | 180 ± 37 | 794 ± 130 |
| 277 | Galβ1-3GlcNAcβ1-3Galβ1-3GlcNAcβ-Sp0 | 513 ± 607 | 1779 ± 1216 |
| 278 | Galβ1-4[Fucα1-3][6OSO3]GlcNAc-Sp0 | 204 ± 40 | 1138 ± 454 |
| 279 | Galβ1-4[Fucα1-3][6OSO3]Glc-Sp0 | 675 ± 303 | 4766 ± 640 |
| 280 | Galβ1-4(Fucα1-3)GlcNAcβ1-3Galβ1-3(Fucα1-4)GlcNAcβ-Sp0 | 140 ± 34 | 562 ± 87 |
| 281 | Galβ1-4GlcNAcβ1-3Galβ1-3GlcNAcβ-Sp0 | 211 ± 44 | 1248 ± 350 |
| 282 | Neu5Acα2-3Galβ1-3GlcNAcβ1-3Galβ1-3GlcNAcβ-Sp0 | 3700 ± 1725 | 570 ± 219 |
| 283 | Neu5Acα2-3Galβ1-4GlcNAcβ1-3Galβ1-3GlcNAcβ-Sp0 | 779 ± 297 | 431 ± 125 |
| 284 | [3OSO3]Galβ1-4[6OSO3]GlcNAcβ-Sp0 | 273 ± 59 | 1021 ± 186 |
| 285 | [3OSO3][4OSO3]Galβ1-4GlcNacβ-SpSp0 | 178 ± 65 | 951 ± 318 |
| 286 | [6OSO3]Galβ1-4[6OSO3]GlcNacβ-Sp0 | 706 ± 97 | 3669 ± 263 |
| 287 | 6-H2PO3Glcβ-Sp10 | 206 ± 66 | 988 ± 259 |
| 288 | Galα1-3(Fucα1-2)Galβ–Sp18 | 600 ± 668 | 1782 ± 1509 |
| 289 | Galα1-3GalNAcα-Sp16 | 535 ± 297 | 1022 ± 125 |
| 290 | Galβ1-3GalNAcα-Sp16 | 111 ± 117 | 399 ± 129 |
| 291 | Galβ1-3(Neu5Acα2-3Galβ1-4(Fucα1-3)GlcNAcβ1-6)GalNAc–Sp14 | 340 ± 43 | 655 ± 212 |
| 292 | Galβ1-3Galβ1-4GlcNAcβ-Sp8 | 746 ± 531 | 1668 ± 902 |
| 293 | Galβ1-4GlcNAcβ1-2Manα1-3(Neu5Acα2-6Galβ1-4GlcNAcβ1-2Manα1-6)Manβ1-4GlcNAcβ1-4GlcNAcβ-Sp12 | 94 ± 15 | 262 ± 38 |
| 294 | Galβ1-4GlcNAcβ1-3(Galβ1-4GlcNAcβ1-6)Galβ1-4GlcNAc-Sp0 | 106 ± 45 | 448 ± 79 |
| 295 | Galβ1-4GlcNAcβ1-3(GlcNAcβ1-6)Galβ1-4GlcNAc-Sp0 | 272 ± 78 | 2402 ± 861 |
| 296 | Galβ1-4GlcNAcα1-6Galβ1-4GlcNAcβ-Sp0 | 86 ± 29 | 318 ± 83 |
| 297 | Galβ1-4GlcNAcβ1-6Galβ1-4GlcNAcβ-Sp0 | 102 ± 21 | 283 ± 65 |
| 298 | GalNAcα-Sp15 | 155 ± 126 | 632 ± 489 |
| 299 | GalNAcα1-3(Fucα1-2)Galβ–Sp18 | 338 ± 103 | 3493 ± 612 |
| 300 | GalNAcβ1-3Galβ-Sp8 | 310 ± 133 | 751 ± 205 |
| 301 | GlcAβ1-3GlcNAcβ-Sp8 | 320 ± 184 | 1027 ± 305 |
| 302 | GlcNAcβ1-2Manα1-3(Neu5Acα2-6Galβ1-4GlcNAcβ1-2Manα1-6)Manβ1-4GlcNAcβ1-4GlcNAcβ-Sp12 | 225 ± 110 | 1841 ± 555 |
| 303 | GlcNAcβ1-2Manα1-3(GlcNAcβ1-2Manα1-6)Manβ1-4GlcNAcβ1-4GlcNAcβ-Sp12 | 218 ± 47 | 1022 ± 172 |
| 304 | GlcNAcβ1-3Man-Sp10 | 466 ± 166 | 2733 ± 1837 |
| 305 | GlcNAcβ1-4GlcNAcβ-Sp10 | 93 ± 35 | 600 ± 288 |
| 306 | GlcNAcβ1-4GlcNAcβ-Sp12 | 657 ± 655 | 2619 ± 603 |
| 307 | HOOC(CH3)CH-3-O-GlcNAcβ1-4GlcNAcβ-Sp10 | 136 ± 36 | 490 ± 63 |
| 308 | Manα1-3(Manα1-6)Manβ1-4GlcNAcβ1-4GlcNAcβ-Sp12 | 155 ± 31 | 380 ± 62 |
| 309 | Manα1-6Manβ-Sp10 | 332 ± 151 | 2258 ± 491 |
| 310 | Manα1-6(Manα1-3)Manα1-6(Manα1-3)Manβ-Sp10 | 1000 ± 468 | 768 ± 303 |
| 311 | Manα1-2Manα1-2Manα1-3(Manα1-2Manα1-6(Manα1-3)Manα1-6)Manα-Sp9 | 27296 ± 1732 | 380 ± 135 |
| 312 | Manα1-2Manα1-2Manα1-3(Manα1-2Manα1-6(Manα1-2Manα1-3)Manα1-6)Manα-Sp9 | 31775 ± 1189 | 375 ± 327 |
| 313 | Neu5Acα2-3Galβ1-3(Neu5Acα2-3Galβ1-4GlcNAcβ1-6)GalNAc–Sp14 | 329 ± 145 | 1250 ± 110 |
| 314 | Neu5Acα2-3Galβ1-3(Neu5Acα2-6)GalNAc-Sp14 | 1016 ± 194 | 1287 ± 216 |
| 315 | Neu5Acα2-3Galβ1-3GalNAc–Sp14 | 1471 ± 993 | 7713 ± 3532 |
| 316 | Neu5Acα2-3Galβ1-4GlcNAcβ1-2Manα1-3(Neu5Acα2-6Galβ1-4GlcNAcβ1-2Manα1-6)Manβ1-4GlcNAcβ1-4GlcNAcβ-Sp12 | 100 ± 40 | 267 ± 56 |
| 317 | Neu5Acα2-6Galβ1-4GlcNAcβ1-2Manα1-3(Galβ1-4GlcNAcβ1-2Manα1-6)Manβ1-4GlcNAcβ1-4GlcNAcβ-Sp12 | 643 ± 427 | 5625 ± 1218 |
| 318 | Neu5Acα2-6Galβ1-4GlcNAcβ1-2Manα1-3(GlcNAcβ1-2Manα1-6)Manβ1-4GlcNAcβ1-4GlcNAcβ-Sp12 | 148 ± 64 | 214 ± 40 |
| 319 | Neu5Acα2-6Galβ1-4GlcNAcβ1-2Manα1-3(Neu5Acα2-6Galβ1-4GlcNAcβ1-2Manα1-6)Manβ1-4GlcNAcβ1-4GlcNAcβ-N(LT)AVL | 302 ± 139 | 562 ± 199 |
| 320 | Fucα1-2Galβ1-3GalNAcα-Sp14 | 1184 ± 501 | 5886 ± 1752 |
| 321 | Galβ1-3(Neu5Acα2-6)GalNAcα-Sp14 | 2281 ± 238 | 856 ± 445 |
| 322 | Galβ1-4GlcNAcβ1-3GalNAc-Sp14 | 755 ± 311 | 4648 ± 1054 |
| 323 | NeuAc(9Ac)α2-3Galβ1-4GlcNAcβ-Sp0 | 192 ± 47 | 1407 ± 171 |
| 324 | NeuAc(9Ac)α2-3Galβ1-3GlcNAcβ-Sp0 | 108 ± 121 | 455 ± 53 |
| 325 | NeuAcα2-6Galβ1-4GlcNAcβ1-3Galβ1-3GlcNAcβ-Sp0 | 792 ± 374 | 1650 ± 65 |
| 326 | NeuAcα2-3Galβ1-3(Fucα1-4)GlcNAcβ1-3Galβ1-3(Fucα1-4)GlcNAcβ-Sp0 | 12062 ± 3423 | 771 ± 138 |
| 327 | NeuAcα2-6Galβ1-4GlcNAcβ1-3Galβ1-4GlcNAcβ1-3Galβ1-4GlcNAcβ-Sp0 | 688 ± 545 | 3650 ± 2373 |
| 328 | Galα1-4Galβ1-4GlcNAcβ1-3Galβ1-4Glcβ-Sp0 | 109 ± 66 | 928 ± 143 |
| 329 | GalNAcβ1-3Galα1-4Galβ1-4GlcNAcβ1-3Galβ1-4Glcβ-Sp0 | 397 ± 193 | 3540 ± 286 |
| 330 | GalNAcα1-3(Fucα1-2)Galβ1-4GlcNAcβ1-3Galβ1-4GlcNAcβ-Sp0 | 175 ± 42 | 598 ± 135 |
| 331 | GalNAcα1-3(Fucα1-2)Galβ1-4GlcNAcβ1-3Galβ1-4GlcNAcβ1-3Galβ1-4GlcNAcβ-Sp0 | 1810 ± 404 | 6276 ± 894 |
| 332 | (Neu5Acα2-3-Galβ1-3)(((Neu5Acα2-3-Galβ1-4(Fucα1-3))GlcNAcβ1-6)GalNAc–Sp14 | 871 ± 248 | 1345 ± 117 |
| 333 | GlcNAcα1-4Galβ1-4GlcNAcβ1-3Galβ1-4GlcNAcβ1-3Galβ1-4GlcNAcβ-Sp0 | 328 ± 202 | 2237 ± 1187 |
| 334 | GlcNAcα1-4Galβ1-4GlcNAcβ-Sp0 | 1279 ± 694 | 1561 ± 426 |
| 335 | GlcNAcα1-4Galβ1-3GlcNAcβ-Sp0 | 377 ± 181 | 1416 ± 226 |
| 336 | GlcNAcα1-4Galβ1-4GlcNAcβ1-3Galβ1-4Glcβ-Sp0 | 535 ± 169 | 462 ± 115 |
| 337 | GlcNAcα1-4Galβ1-4GlcNAcβ1-3Galβ1-4(Fucα1-3)GlcNAcβ1-3Galβ1-4(Fucα1-3)GlcNAcβ-Sp0 | 10141 ± 3862 | 5217 ± 312 |
| 338 | GlcNAcα1-4Galβ1-4GlcNAcβ1-3Galβ1-4GlcNAcβ-Sp0 | 710 ± 378 | 988 ± 158 |
| 339 | GlcNAcα1-4Galβ1-3GalNAc-Sp14 | 1166 ± 209 | 4439 ± 1389 |
| 340 | Manα1-3(Neu5Acα2-6Galβ1-4GlcNAcβ1-2Manα1-6)Manβ1-4GlcNAcβ1-4GlcNAc-Sp12 | 176 ± 53 | 323 ± 74 |
| 341 | Neu5Acα2-6Galβ1-4GlcNAcβ1-2Manα1-3(Manα1-6)Manβ1-4GlcNAcβ1-4GlcNAc-Sp12 | 295 ± 152 | 3551 ± 2062 |
| 342 | Neu5Acα2-6Galβ1-4GlcNAcβ1-2Manα1-6Manβ1-4GlcNAcβ1-4GlcNAc-Sp12 | 97 ± 21 | 249 ± 49 |
| 343 | Neu5Acα2-6Galβ1-4GlcNAcβ1-2Manα1-3Manβ1-4GlcNAcβ1-4GlcNAc-Sp12 | 555 ± 390 | 6224 ± 2364 |
| 344 | Galβ1-4GlcNAcβ1-2Manα1-3Manβ1-4GlcNAcβ1-4GlcNAc-Sp12 | 131 ± 54 | 350 ± 52 |
| 345 | Galβ1-4GlcNAcβ1-2Manα1-6Manβ1-4GlcNAcβ1-4GlcNAc-Sp12 | 454 ± 143 | 2034 ± 348 |
| 346 | Galβ1-4GlcNAcβ1-2Manα1-3(Manα1-6)Manβ1-4GlcNAcβ1-4GlcNAcβ-Sp12 | 370 ± 409 | 609 ± 231 |
| 347 | GlcNAcβ1-2Manα1-3(GlcNAcβ1-2Manα1-6)Manβ1-4GlcNAcβ1-4(Fucα1-6)GlcNAcβ-Sp22 | 2887 ± 407 | 1183 ± 277 |
| 348 | Galβ1-4GlcNAcβ1-2Manα1-3(Galβ1-4GlcNAcβ1-2Manα1-6)Manβ1-4GlcNAcβ1-4(Fucα1-6)GlcNAcβ-Sp22 | 1162 ± 1269 | 261 ± 46 |
| 349 | Galβ1-3GlcNAcβ1-2Manα1-3(Galβ1-3GlcNAcβ1-2Manα1-6)Manβ1-4GlcNAcβ1-4(Fucα1-6)GlcNacβ-Sp22 | 5850 ± 5384 | 836 ± 171 |
| 350 | Galβ1-3(Fucα1-4)GlcNAcβ1-2Manα1-3[Galβ1-3(Fucα1-4)GlcNAcβ1-2Manα1-6]Manβ1-4GlcNAcβ1-4GlcNAcβ-Sp19 | 5688 ± 915 | 188 ± 112 |
| 351 | (6SO3)GlcNAcβ1-3Gal β1-4GlcNAc-β-Sp0 | 217 ± 112 | 406 ± 77 |
| 352 | KDNα2-3Galβ1-4(Fucα1-3)GlcNAc-Sp0 | 529 ± 287 | 2624 ± 1286 |
| 353 | KDNα2-6Galβ1-4GlcNAc-Sp0 | 290 ± 173 | 539 ± 132 |
| 354 | KDNα2-3Galβ1-4Glc-Sp0 | 612 ± 363 | 4258 ± 729 |
| 355 | KDNα2-3Galβ1-3GalNAcα-Sp14 | 282 ± 59 | 1093 ± 205 |
| 356 | Fucα1-2Galβ1-3GlcNAcβ1-2Manα1-3(Fucα1-2Galβ1-3GlcNAcβ1-2Manα1-6)Manβ1-4GlcNAcβ1-4GlcNAcβ-Sp20 | 6842 ± 751 | 2798 ± 837 |
| 357 | Fucα1-2Galβ1-4GlcNAcβ1-2Manα1-3(Fucα1-2Galβ1-4GlcNAcβ1-2Manα1-6)Manβ1-4GlcNAcβ1-4GlcNAcβ-Sp20 | 10513 ± 5173 | 282 ± 81 |
| 358 | Fucα1-2Galβ1-4(Fucα1-3)GlcNAcβ1-2Manα1-3[Fucα1-2Galβ1-4(Fucα1-3)GlcNAcβ1-2Manα1-6]Manβ1-4GlcNAcβ1-4GlcNAβ-Sp20 | 8348 ± 986 | 1049 ± 136 |
| 359 | Galα1-3Galβ1-4GlcNAcβ1-2Manα1-3(Galα1-3Galβ1-4GlcNAcβ1-2Manα1-6)Manβ1-4GlcNAcβ1-4GlcNAcβ-Sp20 | 408 ± 383 | 250 ± 51 |
| 360 | Manα1-3(Galβ1-4GlcNAcβ1-2Manα1-6)Manβ1-4GlcNAcβ1-4GlcNAcβ-Sp12 | 547 ± 753 | 1110 ± 188 |
| 361 | Galβ1-3(Fucα1-4)GlcNAcβ1-2Manα1-3[Galβ1-3(Fucα1-4)GlcNAcβ1-2Manα1-6]Manβ1-4GlcNAcβ1-4(Fucα1-6)GlcNAcβ-Sp22 | 26003 ± 2473 | 454 ± 62 |
| 362 | Neu5Acα2-6GlcNAcβ1-4GlcNAc-Sp21 | 1033 ± 853 | 3468 ± 2301 |
| 363 | Neu5Acα2-6GlcNAcβ1-4GlcNAcβ1-4GlcNAc-Sp21 | 176 ± 42 | 457 ± 69 |
| 364 | Fucα1-2Galβ1-3GlcNAcβ1-3[Galβ1-4(Fucα1-3)GlcNAcβ1-6]Galβ1-4Glc-Sp21 | 565 ± 342 | 3386 ± 2132 |
| 365 | Galβ1-4GlcNAcβ1-2(Galβ1-4GlcNAcβ1-4)Manα1-3[Galβ1-4GlcNAcβ1-2Manα1-6]Manβ1-4GlcNAcβ1-4GlcNAc-Sp21 | 177 ± 74 | 503 ± 46 |
| 366 | GalNAcα1-3(Fucα1-2)Galβ1-4GlcNAcβ1-2Manα1-3[GalNAcα1-3(Fucα1-2)Galβ1-4GlcNAcβ1-2Manα1-6]Manβ1-4GlcNAcβ1-4GlcNAcβ-Sp20 | 1881 ± 485 | 3789 ± 1364 |
| 367 | Galα1-3(Fucα1-2)Galβ1-4GlcNAcβ1-2Manα1-3[Galα1-3(Fucα1-2)Galβ1-4GlcNAcβ1-2Manα1-6]Manβ1-4GlcNAcβ1-4GlcNAcβ-Sp20 | 2006 ± 227 | 323 ± 21 |
| 368 | Galα1-3Galβ1-4(Fucα1-3)GlcNAcβ1-2Manα1-3[Galα1-3Galβ1-4(Fucα1-3)GlcNAcβ1-2Manα1-6]Manβ1-4GlcNAcβ1-4GlcNAcβ-Sp20 | 921 ± 152 | 2754 ± 634 |
| 369 | GalNAcα1-3(Fucα1-2)Galβ1-3GlcNAcβ1-2Manα1-3[GalNAcα1-3(Fucα1-2)Galβ1-3GlcNAcβ1-2Manα1-6]Manβ1-4GlcNAcβ1-4GlcNAcβ-Sp20 | 3293 ± 1544 | 282 ± 192 |
| 370 | Galα1-3(Fucα1-2)Galβ1-3GlcNAcβ1-2Manα1-3[Galα1-3(Fucα1-2)Galβ1-3GlcNAcβ1-2Manα1-6]Manβ1-4GlcNAcβ1-4GlcNAcβ-Sp20 | 10295 ± 681 | 328 ± 142 |
| 371 | Fucα1-2Galβ1-3(Fucα1-4)GlcNAcβ1-2Manα1-3[Fucα1-2Galβ1-3(Fucα1-4)GlcNAcβ1-2Manα1-6]Manβ1-4GlcNAcβ1-4GlcNAcβ-Sp19 | 4237 ± 700 | 1016 ± 218 |
| 372 | NeuAcα2-3Galβ1-4GlcNAcβ1-3GalNAc-Sp14 | 459 ± 418 | 1034 ± 241 |
| 373 | NeuAcα2-6Galβ1-4GlcNAcβ1-3GalNAc-Sp14 | 1299 ± 645 | 730 ± 343 |
| 374 | Fucα1-3[NeuAcα2-3Galβ1-4]GlcNAcβ1-3GalNAc-Sp14 | 7124 ± 875 | 1411 ± 421 |
| 375 | GalNAcβ1-4GlcNAcβ1-2Manα1-6(GalNAcβ1-4GlcNAcβ1-2Manα1-6)Manβ1-4GlcNAcβ1-4GlcNAc-Sp12 | 414 ± 230 | 523 ± 217 |
| 376 | Galβ1-3GalNAcα1-3(Fucα1-2)Galβ1-4Glc-Sp14 | 129 ± 39 | 291 ± 125 |
| 377 | Galβ1-3GalNAcα1-3(Fucα1-2)Galβ1-4GlcNAc-Sp14 | 272 ± 124 | 361 ± 128 |

**Additional File 1. Glycan array data for CL-K1 in the presence and absence of Ca^2+^.** Data are relative fluorescence ± SE from six replicates. The best ligands, with binding values >10000 are *shaded*.
